# From retina to brain: retinal dysfunction and dopaminergic neurodegeneration in *Drosophila* models of Parkinson’s disease

**DOI:** 10.3389/fcell.2026.1929128

**Published:** 2026-09-23

**Authors:** Justyna Kadłuczka, Jakub Dymek, Elżbieta Pyza, Milena Damulewicz

**Affiliations:** 1 Department of Cell Biology and Imaging, Institute of Zoology and Biomedical Research, Faculty of Biology, Jagiellonian University, Kraków, Poland; 2 Doctoral School of Exact and Natural Sciences, Jagiellonian University, Krakow, Poland

**Keywords:** dopaminergic (DA) neuron, *Drosophila melanogaster*, parkin, retina, synuclein alpha (SNCA)

## Abstract

Parkinson’s disease (PD) is characterized by the progressive loss of dopaminergic neurons within the substantia nigra pars compacta. Although PD is primarily defined as a motor disorder, prodromal non-motor symptoms - such as circadian clock desynchronization and sleep disturbances - can emerge years before clinical diagnosis. Increasing evidence suggests that these non-motor manifestations, particularly circadian disruption, may contribute to disease progression. However, it remains unclear whether circadian dysfunction actively drives neurodegeneration or arises as a consequence of ongoing neuronal loss. In this study, we employed genetic models of PD in *Drosophila melanogaster* to investigate these relationships. Specifically, we examined the effects of *parkin* (*park*) silencing and neuronal expression of human α-Synuclein on photoreceptor morphology and function. Furthermore, we assessed whether mitophagy impairment or synucleinopathy confined to the visual system can influence the onset and progression of PD-related phenotypes. Given that sleep fragmentation is a common feature in PD patients, we also analyzed sleep patterns in flies, focusing on sleep duration. In parallel, we evaluated dopaminergic neuron degeneration, with particular emphasis on the protocerebral anterior medial (PAM) cluster, which plays a critical role in the regulation of sleep and locomotor activity. Taken together, our findings highlight the importance of elucidating the interplay between retinal cells disruption and neurodegeneration in deeper parts of the brain in Parkinson’s disease.

## Introduction

Parkinson’s disease (PD) is a progressive neurodegenerative disorder primarily affecting motor function. The etiology of PD is complex and multifactorial; most cases are considered idiopathic, meaning that the exact cause is unknown and likely involves a combination of genetic and environmental factors. Only a subset of cases has a clear genetic origin, with pathogenic variants identified in approximately 5%–15% of patients. Among these, mutations in genes such as *SNCA* (encoding α-synuclein) and *PARK2* (encoding parkin) have been strongly associated with familial forms of the disease and have provided key insights into its molecular pathogenesis.

α-Synuclein (α-Syn), encoded by the *SNCA* gene, is a small protein expressed in neurons of both the central and peripheral nervous systems, as well as in other tissues (Burré et al., 2018). α-Syn is predominantly localized at presynaptic terminals, where it interacts with proteins involved in neurotransmitter release and reuptake (Burré et al., 2010). In addition, α-Syn plays an important role in sensing and stabilizing highly curved membranes (Shen et al., 2012) and in regulating the synaptic vesicle pool and vesicular trafficking (Bridi and Hirth, 2018). In its native state, α-Syn exists predominantly as a monomer; however, it can also adopt a folded tetrameric conformation (Bartels et al., 2011). These two forms coexist under physiological conditions, but an imbalance in the tetramer-to-monomer ratio promotes the transition toward a pathological β-sheet–rich conformation, thereby facilitating the recruitment of additional monomers and leading to the formation of oligomers and amyloid fibrils (Lautenschläger et al., 2018). The E46K mutation, which substitutes glutamic acid for lysine, further enhances α-Syn’s propensity to aggregate and is associated with the development of synucleinopathies (Zarranz et al., 2004; Zhao et al., 2020). The initial step of aggregation involves interactions between the positively charged N-terminus and the negatively charged C-terminal region of α-Syn, triggering primary nucleation (Kumari et al., 2021). This process enhances subsequent protein aggregation through secondary nucleation mechanisms. As a consequence, α-Syn aggregation disrupts neuronal homeostasis by inducing membrane permeabilization, elevating basal cytosolic Ca^2+^ levels, increasing stimulus-evoked Ca^2+^ influx, and delaying Ca^2+^ clearance in neurons (Virdi et al., 2022). Pathological α-Syn binds to mitochondria, disrupting cellular respiration and energy production (Wang et al., 2019), thereby increasing oxidative stress. In addition, protein aggregation impairs proteostasis and autophagic pathways, further contributing to oxidative stress. Extracellular α-Syn can be taken up by glial cells, leading to their activation and promoting neuroinflammation (Wang et al., 2023).

Parkin functions as a stress-inducible E3 ubiquitin ligase that recognizes misfolded proteins and damaged mitochondria and targets them for degradation, thereby preventing cell death. It plays a key role in regulating mitophagy in cooperation with PINK1 (Pickrell and Youle, 2015; Whitworth and Pallanck, 2017). Moreover, parkin is involved in the selective turnover of components of the mitochondrial respiratory chain (Vincow et al., 2013). Mutations in *PARK2* or dysfunction of parkin impair mitochondrial quality control, resulting in increased oxidative stress and activation of apoptotic pathways. Under conditions of moderate stress, however, parkin can promote cell survival by activating pro-survival signaling pathways, including the NF-κB pathway, and by inhibiting caspase-dependent apoptosis (Müller-Rischart et al., 2013).

The retina is a specialized part of the nervous system; however, it remains anatomically and functionally separated from the brain. Despite this separation, retinal dysfunction can influence brain activity, as photoreceptors constitute the first stage of visual signal processing and directly project to central brain structures. Therefore, alterations in photoreceptor function may propagate along visual pathways, ultimately affecting brain function and leading to behavioral changes. Visual impairments associated with Parkinson’s disease (PD) have been proposed as early indicators of disease progression, reflecting underlying neurodegenerative processes (Armstrong, 2011).

To investigate whether localized neuronal dysfunction in the retina can influence brain function, we used *Drosophila melanogaster* as a model organism and induced α-Syn expression and *parkin* silencing specifically in photoreceptor cells. Notably, *Drosophila* does not express endogenous α-Syn, allowing us to assess the effects of its ectopic expression in the absence of physiological background function. This transgenic approach enables the direct evaluation of the pathological role of α-Syn in the retina.

Our results demonstrate pronounced alterations in sleep architecture and locomotor activity, suggesting that photoreceptor dysfunction may influence central neural circuits. These behavioral phenotypes may be attributable to dysfunction or degeneration of dopaminergic neurons, which are critical regulators of both motor activity and circadian behavior. Notably, manipulation of different photoreceptor subtypes reduced the number of dopaminergic neurons within the PAM cluster.

## Results

### Mitophagy disruption model

#### Silencing of *park* alters lifespan and climbing ability

*Drosophila parkin* null mutants exhibit reduced lifespan, locomotor defects, and male sterility (Greene et al., 2003). To investigate whether disrupted mitophagy in the visual system may contribute to the development of PD, we silenced the *park* gene (Supplementary Figure S1) specifically in photoreceptors using *GMR-Gal4* as a driver line (Supplementary Figure S2). Silencing of the *park* gene resulted in a reduced percentage of surviving flies (Figure 1A). To assess general fitness, a negative geotaxis assay was performed. *GMR > parkRNAi* flies showed a non-significant trend towards decreased climbing ability after 30 days (Figure 1B). We also used *Rh1-Gal4*, *Rh3-Gal4* and *Rh6-Gal4* flies to knock-down *park* in a specific photoreceptor type. Rhodopsin-1 is expressed in R1-6, Rhodopsin-3 in R7, whereas Rhodopsin-6 in R8 (Earl and Britt, 2006). Neither *Rh1 > parkRNAi* (Figure 1C) nor *Rh3 > parkRNAi* (Figure 1D) flies showed alterations in climbing ability. For *Rh6 > parkRNAi,* we observed a non-significant trend towards decreased general fitness in aged flies, 30 and 40 days of age (Figure 1E).

**FIGURE 1:**
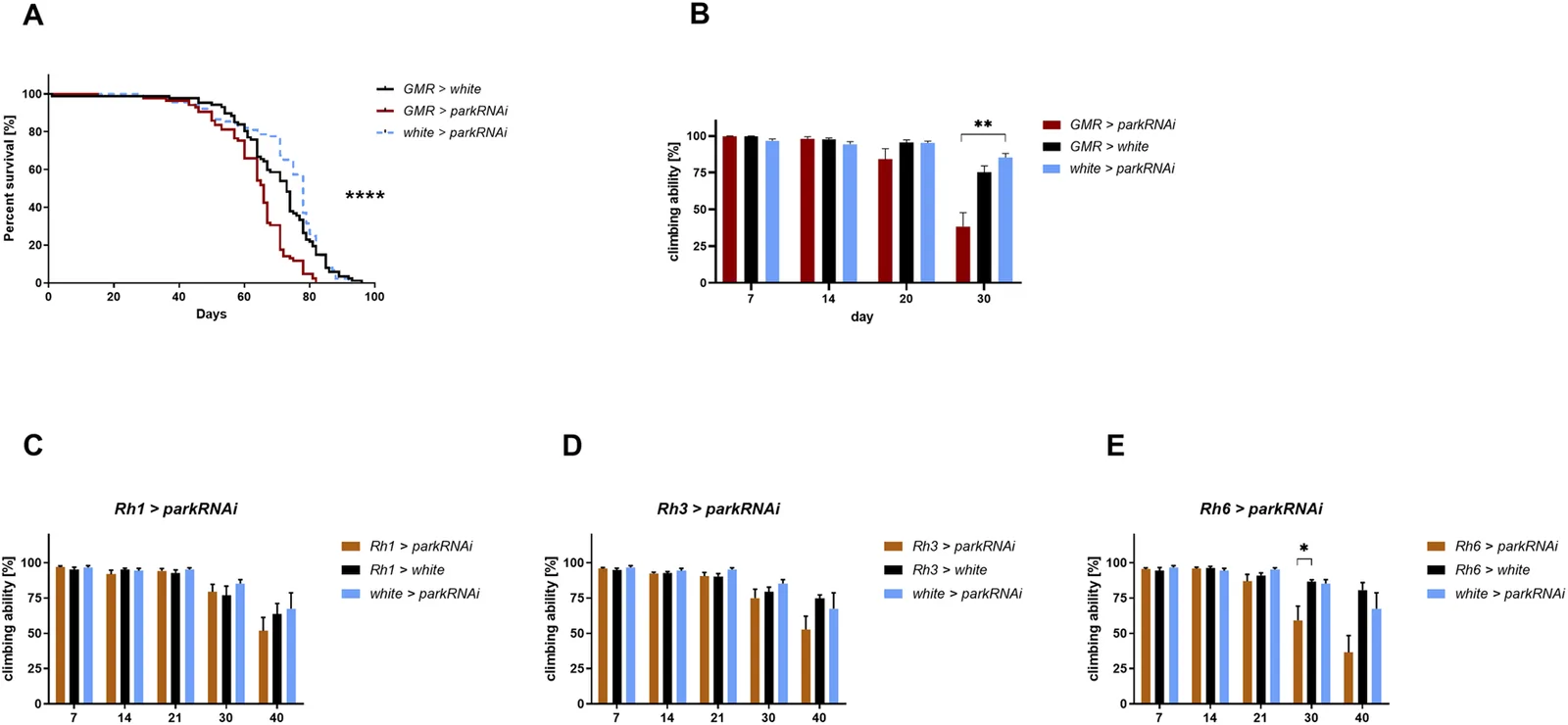
Survival and climbing assay of *Drosophila* adult flies. Silencing of *park* significantly reduced lifespan of the *GMR > parkRNAi* flies compared to the controls **(A)**. ****p < 0.0001 (Log-rank test). The experiment was repeated 3 times, with a total of n = 85 GMR *> parkRNAi* flies, n = 87 GMR *> white* flies, and n = 89 *white > parkRNAi* flies. A non-significant trend towards decreased climbing ability was observed in aged *GMR > parkRNAi* flies **(B)**. Effect of *park* silencing in the specific photoreceptor cells on climbing ability **(C–E)**. No significant changes in general fitness of the flies were observed, although a non-significant trend towards decreased climbing ability in aged (30 and 40-day-old) *Rh6 > parkRNAi* flies was noted **(E)**. The experiment was repeated 6–10 times. Results with error bars are means ± SEM. **p < 0,01 (Kruskal–Wallis test with Dunn’s multiple comparison test).

#### *Park* silencing affected daytime and nighttime sleep

Although PD is primarily described as a motor disorder, prodromal symptoms such as sleep disturbances may occur years before diagnosis (Sohail et al., 2017). It has been implicated that non-motor symptoms, especially circadian clock disruption, can exacerbate the progression of PD (Yalçin et al., 2025). Moreover, patients with PD experience sleep problems, including insomnia and sleep fragmentation (Gjerstad et al., 2007). Thus, we analyzed sleep parameters such as bouts of sleep during the day and night, daytime and nighttime sleep, as well as sleep patterns in young (2-day-old) and old (30-day-old) flies using the DAM system. Silencing of the *park* in young individuals showed no effect on the sleep architecture (Figures 2A–D). Knowing that Parkinson’s is a progressive disease, we also monitored sleep in old flies. Aged *GMR > parkRNAi* flies exhibited an increased number of sleep episodes during the night (Figure 2F), but not during the day (Figure 2E). Daytime sleep was decreased, whereas no changes in total nighttime sleep were observed (Figure 2G). We observed decreased walking activity in young *GMR > parkRNAi* flies during the day (Supplementary Figure S3B), whereas aged flies exhibited reduced mobility during the day and night (Supplementary Figure S3C,D). Normally, flies gradually increase their activity before lights-on (ZT0) and lights-off (ZT12), a phenomenon known as morning and evening anticipation. The sleep pattern of young *GMR > parkRNAi* flies showed no alterations compared to controls (Figure 2D). Old *GMR > parkRNAi* flies, however, exhibited changes in sleep architecture, as they anticipated the light–dark transition earlier than control flies (Figure 2H). We analyzed daily activity profiles of young and aged flies during LD12:12 conditions (Supplementary Figure S4), as well as period of locomotor activity and morning and evening anticipation indexes (Supplementary Table S1).

**FIGURE 2:**
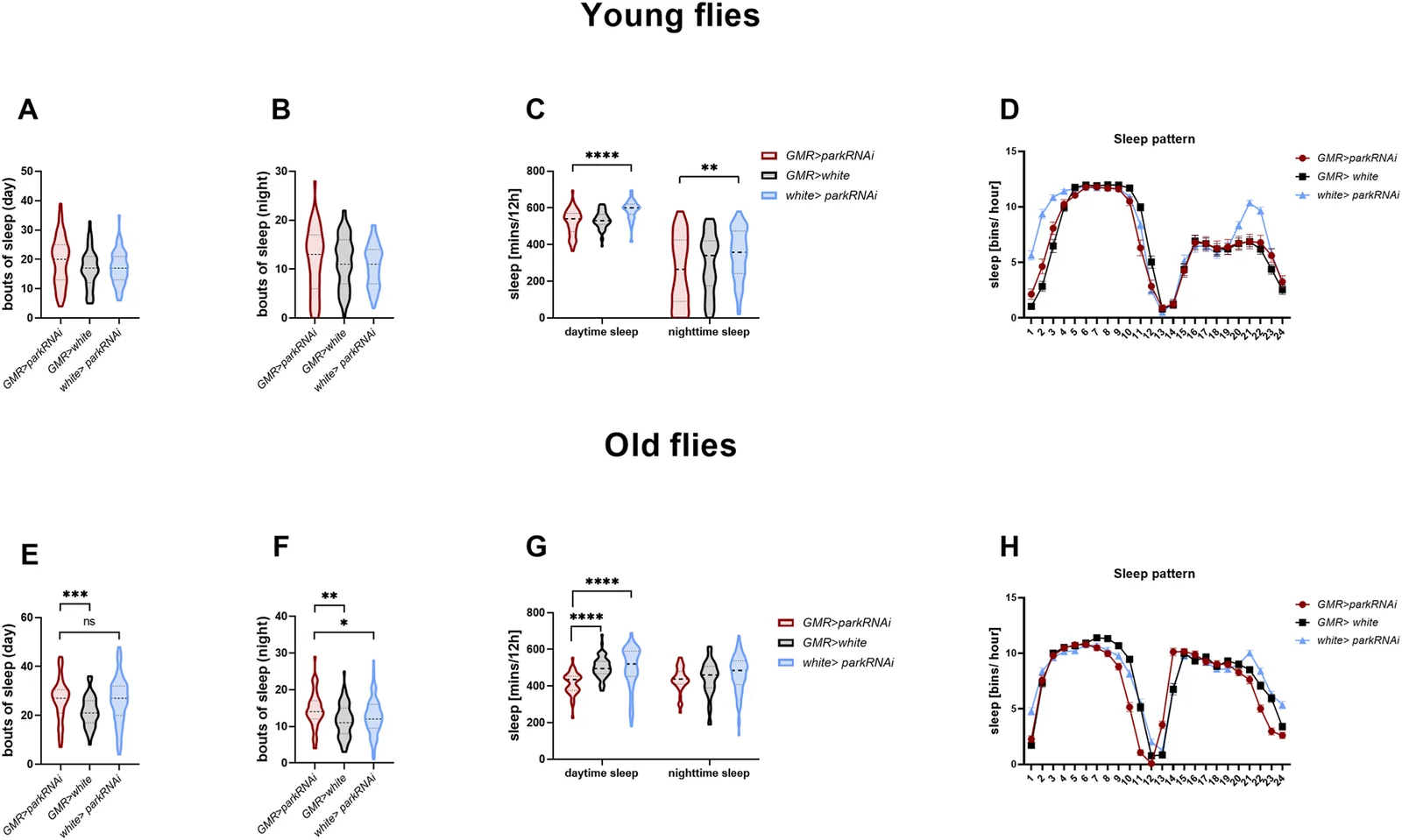
Effect of *park* silencing on sleep homeostasis in young **(A–D)** and old **(E–H)** flies. Young flies showed no alterations in the sleep architecture **(A–D)**. Old flies exhibited sleep fragmentation during the night **(F)** but not during the day **(E)**. Total daytime sleep was decreased in the old flies **(G)** that was reflected in the sleep pattern **(H)**. Data represented in median ± IQR. *p < 0.05, **p < 0.01 ***p < 0.001, ****p < 0.0001 (Kruskal–Wallis test with Dunn’s multiple comparison test).

Interestingly, we observed distinct changes in sleep homeostasis upon silencing the *park* gene in the specific photoreceptor cells. *Rh1 > parkRNAi* flies exhibited a decrease in the number of sleep episodes during the night (Figure 3B), with no effect observed during the day (Figure 3A). Nighttime sleep was increased, whereas daytime sleep remained unchanged (Figure 3C). Silencing of *park* in Rhodopsin-3 expressing cells (*Rh3 > parkRNAi*) led to sleep fragmentation during the day (Figure 3E) but not during the night (Figure 4F). Total daytime sleep was decreased, while the opposite effect was observed for nighttime sleep (Figure 3G). *Rh6 > parkRNAi* flies displayed an increased number of sleep episodes during the night (Figure 3J), with no fragmentation observed during the day (Figure 3I). Total nighttime sleep decreased, whereas daytime sleep was unaffected (Figure 3K). Overall, changes in total sleep duration in the light phase and dark phase were reflected in sleep patterns (Figures 3D,H,L). No changes in walking activity were observed (Supplementary Figure S5). We also analyzed daily activity profiles (Supplementary Figure S6) and calculated period of the rhythm and anticipation indexes (Supplementary Table S1). Here, we observed that *Rh3 > parkRNAi* exhibited a longer period of locomotor activity rhythm (24.29 h) compared to the controls (*Rh3 > white -* 23.84 h, *white > parkRNAi -* 23.79 h; *P* < 0.0001 with both controls). Interestingly *Rh3 > parkRNAi* exhibited significantly lower evening anticipation index compared to the controls (Supplementary Table S1).

**FIGURE 3:**
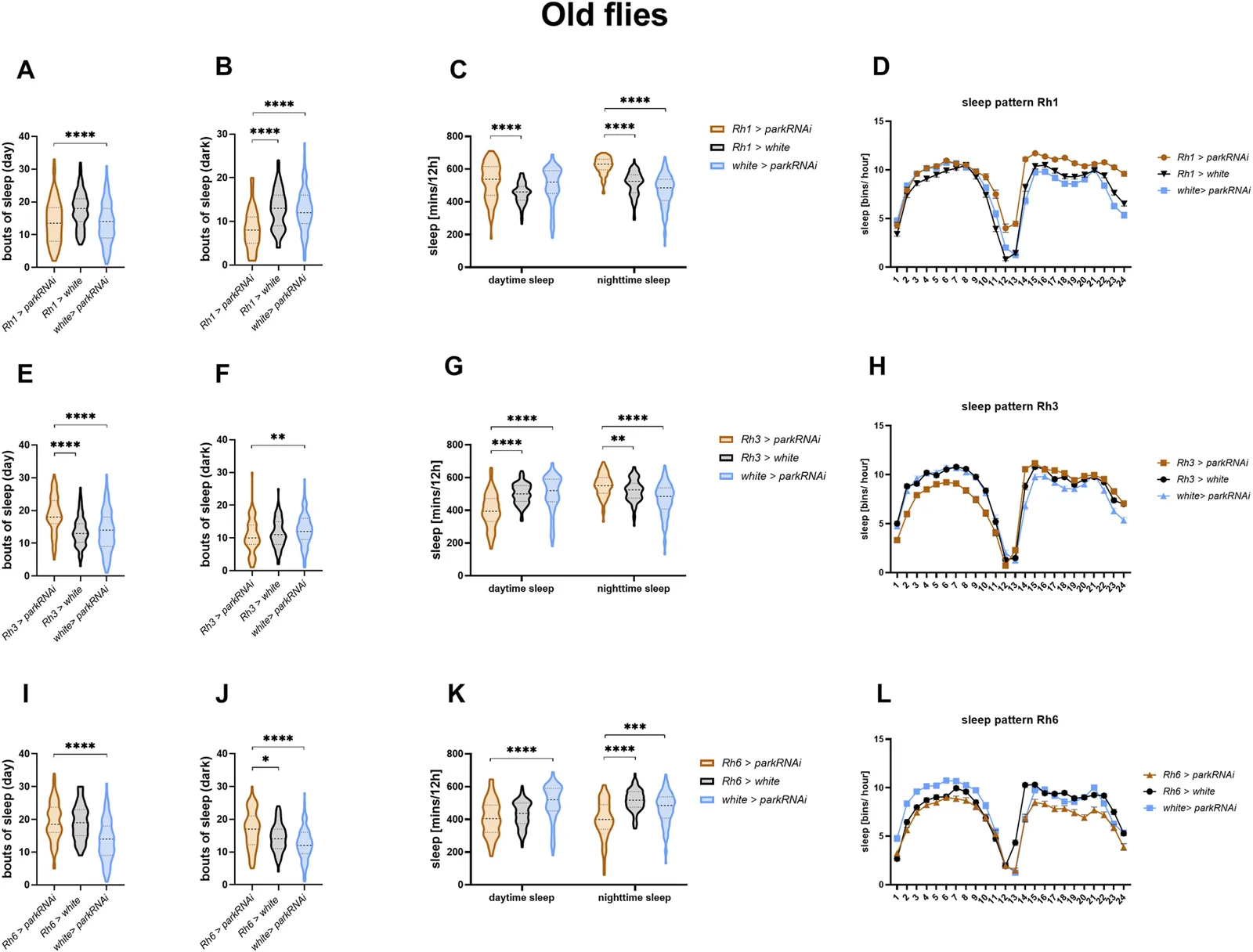
Effect of *park* silencing in the specific photoreceptor cells on sleep homeostasis in the old flies **(A–L)**. Sleep was fragmented during the day only in *Rh3 > parkRNAi* flies **(E)** and during the night in *Rh1 > parkRNAi* **(B)** and *Rh6 > parkRNAi* flies **(J)**. Total daytime sleep was decreased in *Rh3 > parkRNAi* **(G)**. Total nighttime sleep was increased in *Rh1 > parkRNAi* **(C)** and *Rh3 > parkRNAi* **(G)** flies but decreased in *Rh6 > parkRNAi* **(K)**. Changes in sleep duration were reflected in sleep patterns **(D,H,L)**. Data represented in median ± IQR. *p < 0.05, **p < 0.01, ***p < 0.001, ****p < 0.0001 (Kruskal–Wallis test with Dunn’s multiple comparison test).

**FIGURE 4:**
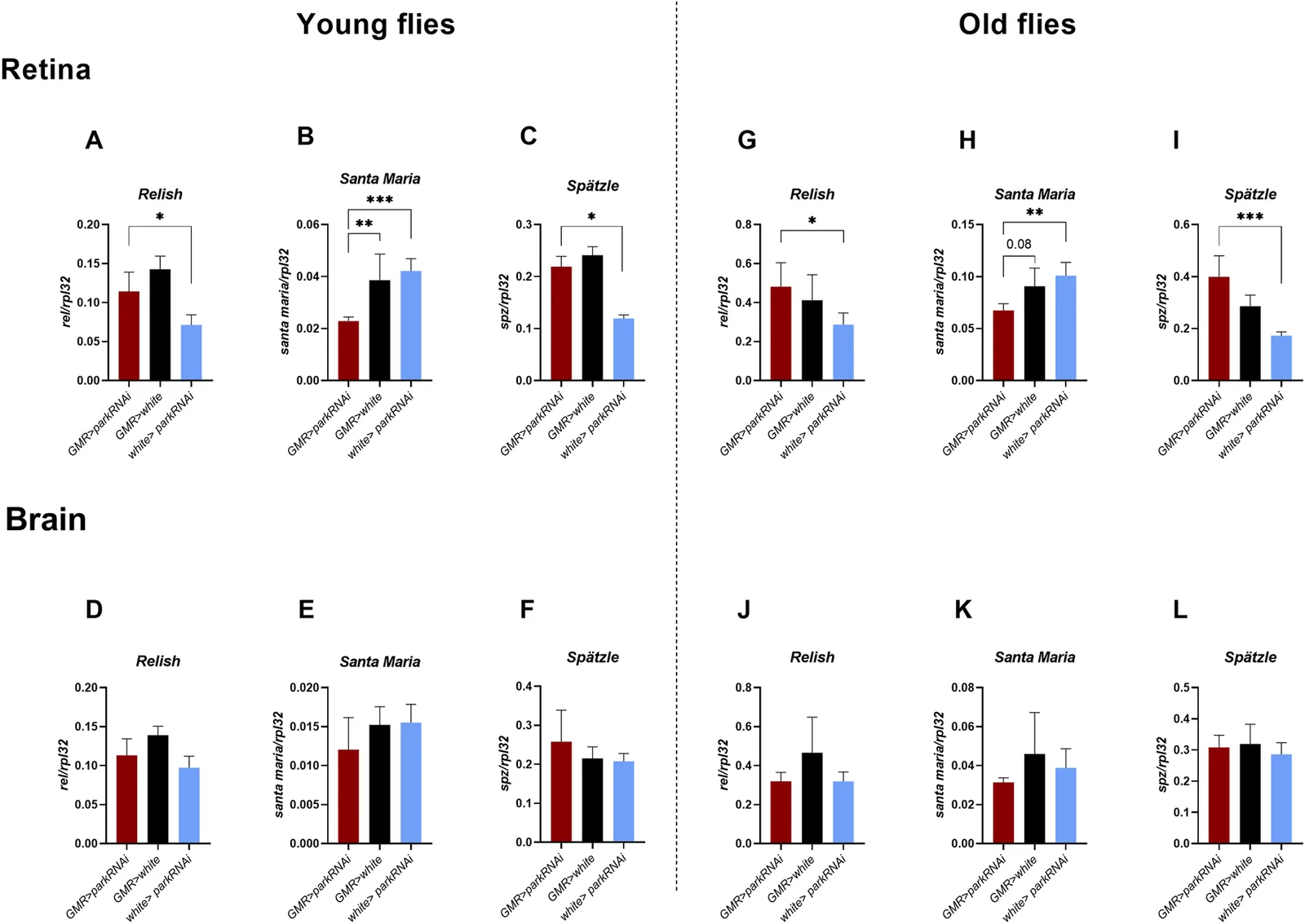
Gene expression analysis in the retina and brain of young **(A–F)** and old flies **(G–L)**. The expression level of *Santa maria* gene was decreased in *GMR > parkRNAi* young flies compared to the controls in the retina **(B)**. No significant changes in the expression levels of *Relish* **(A)** or *Spätzle* **(C)** were observed in the retina. None of the mRNA levels were altered in the brain **(D–F)**. No significant changes in the expression levels of *Relish* **(G)** or *Spätzle* **(I)** were observed in the retina of old flies, although a non-significant trend towards increased expression in *GMR > parkRNAi* flies was noted. Expression level of *Santa Maria* was decreased **(H)**. None of the mRNA levels were altered in the brain **(J–L)**. Results with error bars are means ± SD. *p < 0.05, **p < 0.01, ***p < 0.001 (Kruskal–Wallis test with Dunn’s multiple comparison test).

#### Effect of *park* silencing on inflammation, phototransduction and autophagy

We investigated the expression level of genes involved in immune response, visual function, and autophagy, separately in the retina and brain. The mRNA level of *Santa Maria* in the retina was significantly decreased in young (7-day-old) *GMR > parkRNAi* flies (Figure 4B). No difference in the expression levels of *Relish* and *Spätzle* was observed (Figures 4A,C). In addition, no transcriptional alterations were detected in the brain (Figures 4D–F). Similarly to the young flies, aged (40-day-old) *GMR > parkRNAi* flies exhibited a significant reduction of mRNA level of *Santa Maria* in the retina (Figure 4H). No changes in the expression levels of *Relish* or *Spätzle* were observed in the retina, although a non-significant trend towards increased expression in *GMR > parkRNAi* flies was noted (Figures 4G,I). Moreover, none of the mRNA levels were altered in the brain (Figures 4J–L). These results suggest disruption in phototransduction and phagocytic processes.

#### Assessment of the outer and inner *Drosophila* eye structure

To visualize the morphology of the *Drosophila* eye in young and aged flies, we used scanning electron microscopy (SEM). No alterations were observed in the ommatidial arrangement or the structure of mechanosensory (interommatidial) bristles across any of the genotypes (Figures 5A–F), indicating that external eye morphology remained intact. Moreover, we performed histological staining with toluidine blue on semi-thin sections of the retina to analyze photoreceptor morphology. No changes were detected in rhabdomere structure (Figures 5G–L), which remained arranged in an asymmetric trapezoid at the center of the ommatidia.

**FIGURE 5:**
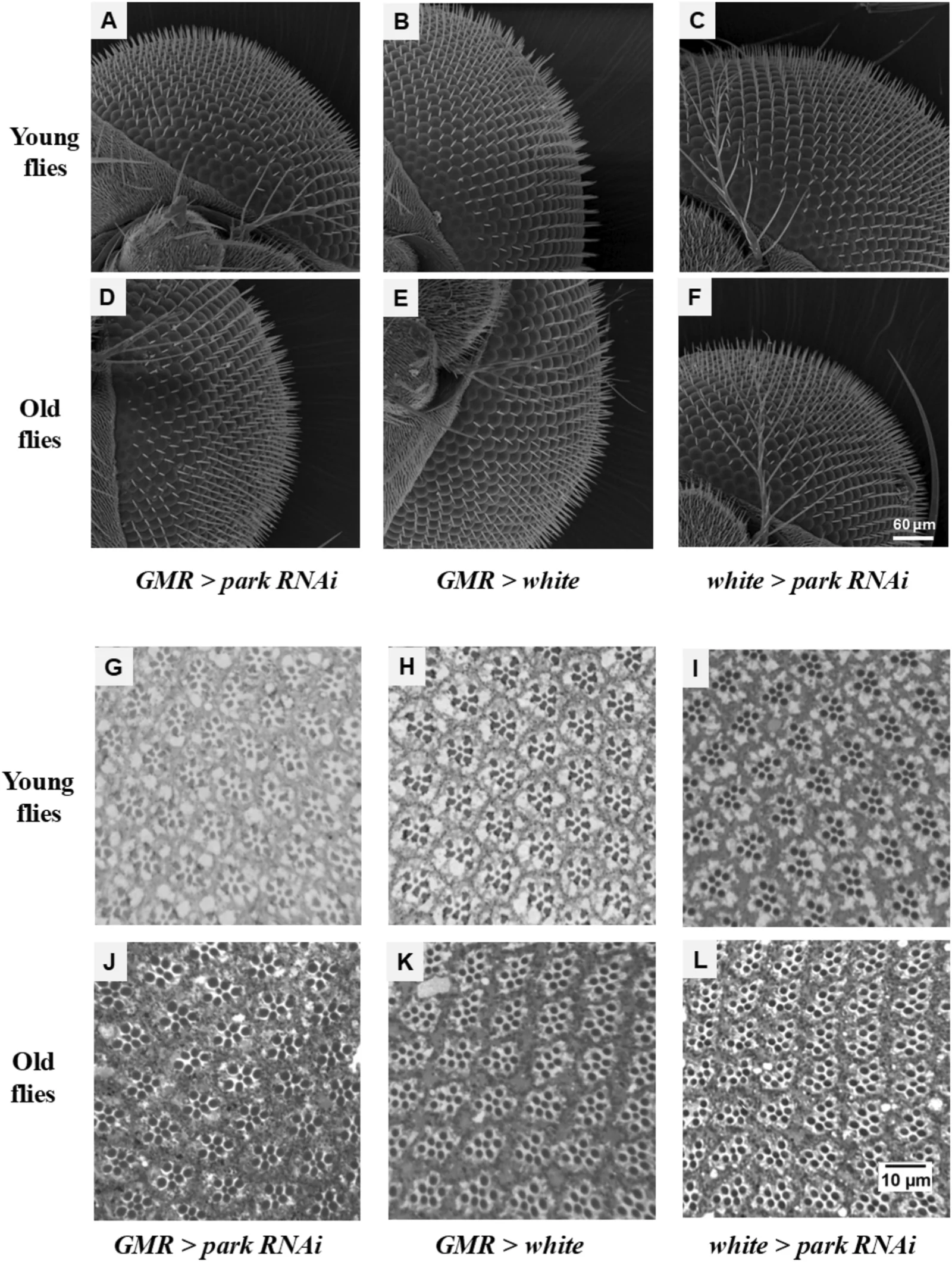
Scanning electron microscopy analysis of the outer eye structure at ×350 magnification **(A–F)**. No effect of *park* silencing was observed on the eye morphology. Images were taken from 10 specimens per group. Cross sections of the retina photoreceptors **(G–L)**. Silencing of *park* showed no effect on the morphology of the photoreceptor cells. Images were captured from at least three flies per group.

#### Silencing of the *park* gene in the visual system did not affect degeneration of PAM neurons

Although the fruit fly brain is small, it shares structural and functional similarities with its human counterpart, including a conserved dopaminergic system. The adult *Drosophila* brain contains approximately ten clusters of dopaminergic neurons that innervate distinct brain regions (Xie et al., 2018). The PAM cluster (Figure 6A) comprises approximately 250 neurons and plays a key role in regulating behaviours such as foraging, sleep–wake cycles, and locomotion (Landayan et al., 2018; Driscoll et al., 2021; Ryu et al., 2022). Importantly, PAM neurons are highly heterogeneous, and distinct subpopulations exhibit varying levels of vulnerability to oxidative stress (Dorcikova et al., 2023). Neurodegeneration in PAM (protocerebral anterior medial), PPL1 (protocerebral anterior lateral 1) and PPM1/2 (protocerebral posterior medial 1/2) clusters has been reported in numerous PD models (Wang C. et al., 2007; Bou Dib et al., 2014; Song et al., 2017). Here, we showed that silencing of the *park* gene in the visual system had no effect on neurodegeneration within PAM cluster (Figure 6B). We observed no changes in the organization of PAM cells between young (Figure 6C) and old (Figure 6D) *GMR > parkRNAi* flies.

**FIGURE 6:**
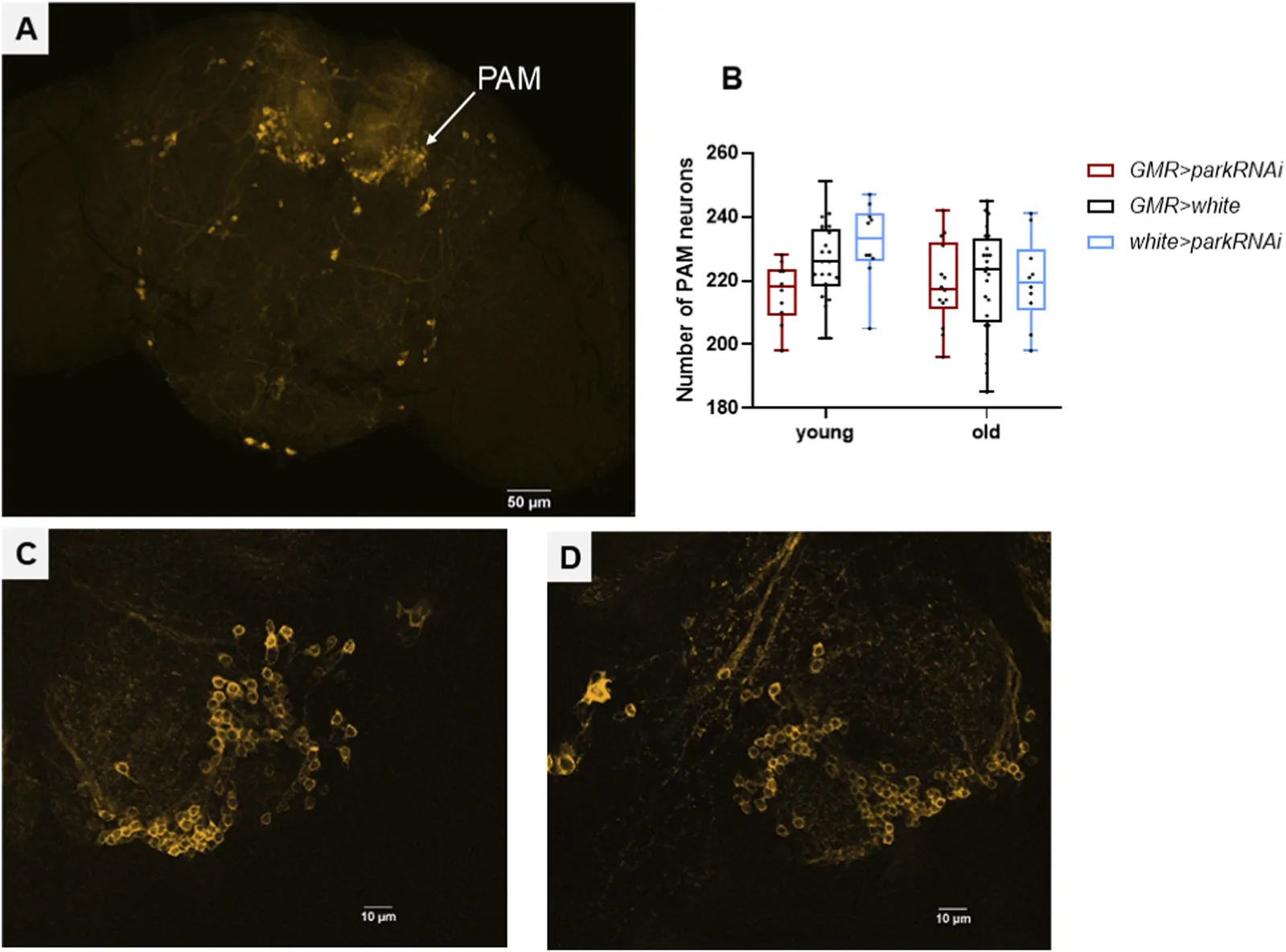
The number of PAM neurons detected by anti-TH immunostaining of young and old flies. Localization of PAM neurons cluster in the adult *Drosophila* brain **(A)**. No effect of *park* silencing on neurodegeneration was observed **(B)**. Representative maximum intensity projection images of PAM neurons in young **(C)** and old **(D)** *GMR > parkRNAi* flies (118 cells in each of the hemispheres). One-way ANOVA with Tukey’s multiple comparison test.

### Synucleinopathy model of PD

#### Expression of mutated α-Syn in the visual system resulted in reduced lifespan

Studies have shown that flies expressing α-Syn exhibit numerous symptoms of PD, such as locomotor dysfunction and progressive loss of dopaminergic neurons (Feany and Bender, 2000). We expressed either wild-type or familial PD-linked (E46K mutation) human *α*-Syn exclusively in photoreceptors. We observed a significant decrease in the lifespan of *GMR > αsynE46K* compared to controls (Figure 7A). Nevertheless, the climbing ability remained unchanged in these flies (Figure 7B). Similarly to the mitophagy disruption model, here we expressed *α*-Syn in a specific photoreceptor type (Figures 7C–E; Supplementary Figure S7). We observed a significant decline in general fitness in *Rh3 > αsynE46K* flies at day 40 (Figure 7D), with no such alterations in *Rh1 > αsynE46K* (Figure 7C) or *Rh6 > αsynE46K* (Figure 7E) flies.

**FIGURE 7:**
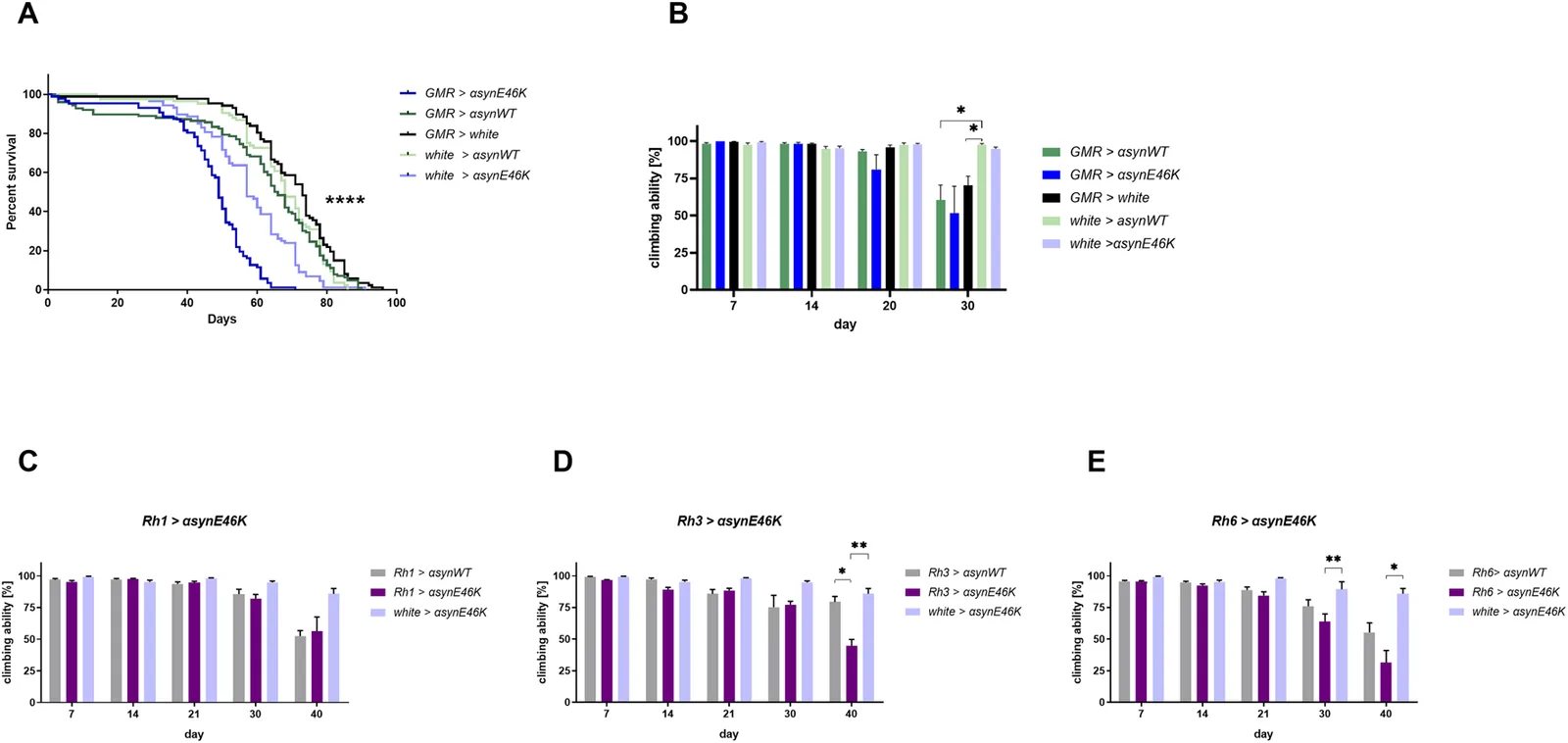
Survival and climbing assay of *Drosophila* adult flies. Expression of mutated α-Syn significantly reduced lifespan of the *GMR > αsynE46K* flies compared to the controls **(A)**. ****p < 0.0001 (Log-rank test). The experiment was repeated 3 times, with a total of n = 87 GMR *> αsynE46K* flies, n = 126 GMR *> αsynWT*, n = 87 GMR *> white* flies, and n = 88 *white > αsynE46K* flies, n = 84 *white > αsynWT*. No effect of mutated α-Syn on climbing ability was observed **(B)**. Expression of mutant α-Syn did not affect climbing ability in aged *Rh1 > αsynE46K* flies **(C)**. In contrast, aged *Rh3 > αsynE46K* flies exhibited impaired general fitness **(D)**, whereas no effect was observed in *Rh6 > αsynE46K* flies **(E)**. The experiment was repeated 5–10 times. Results with error bars are means ± SEM). *p < 0.05, **p < 0.01 (Kruskal–Wallis test with Dunn’s multiple comparison test).

#### Mutated α-Syn alters sleep homeostasis

We tried to investigate how sleep architecture was altered by the expression of α-Syn in the visual system (Figure 8; Supplementary Figure S8). Young *GMR > αsynE46K* flies exhibited no sleep fragmentation either during the day (Figure 8A) or night (Figure 8B). Analyzing the amount of total sleep, we observed a visible decrease in the amount of daytime sleep, but no changes in nighttime sleep were noted (Figures 8C,D). We also sought to examine how sleep is altered in aged flies expressing α-Syn. Old *GMR > αsynE46K* flies showed changes neither in the parameters of sleep episodes (Figures 8E,F) nor in the total amount of sleep (Figures 8G,H). No changes in the walking activity were observed in both young and old flies (Supplementary Figure S9). Similarly to the mitophagy disruption model, we analyzed daily activity profiles of young and aged flies (Supplementary Figure S10), as well as the period of locomotor activity and morning and evening anticipation indexes (Supplementary Table S1). No changes in those parameters were observed.

**FIGURE 8:**
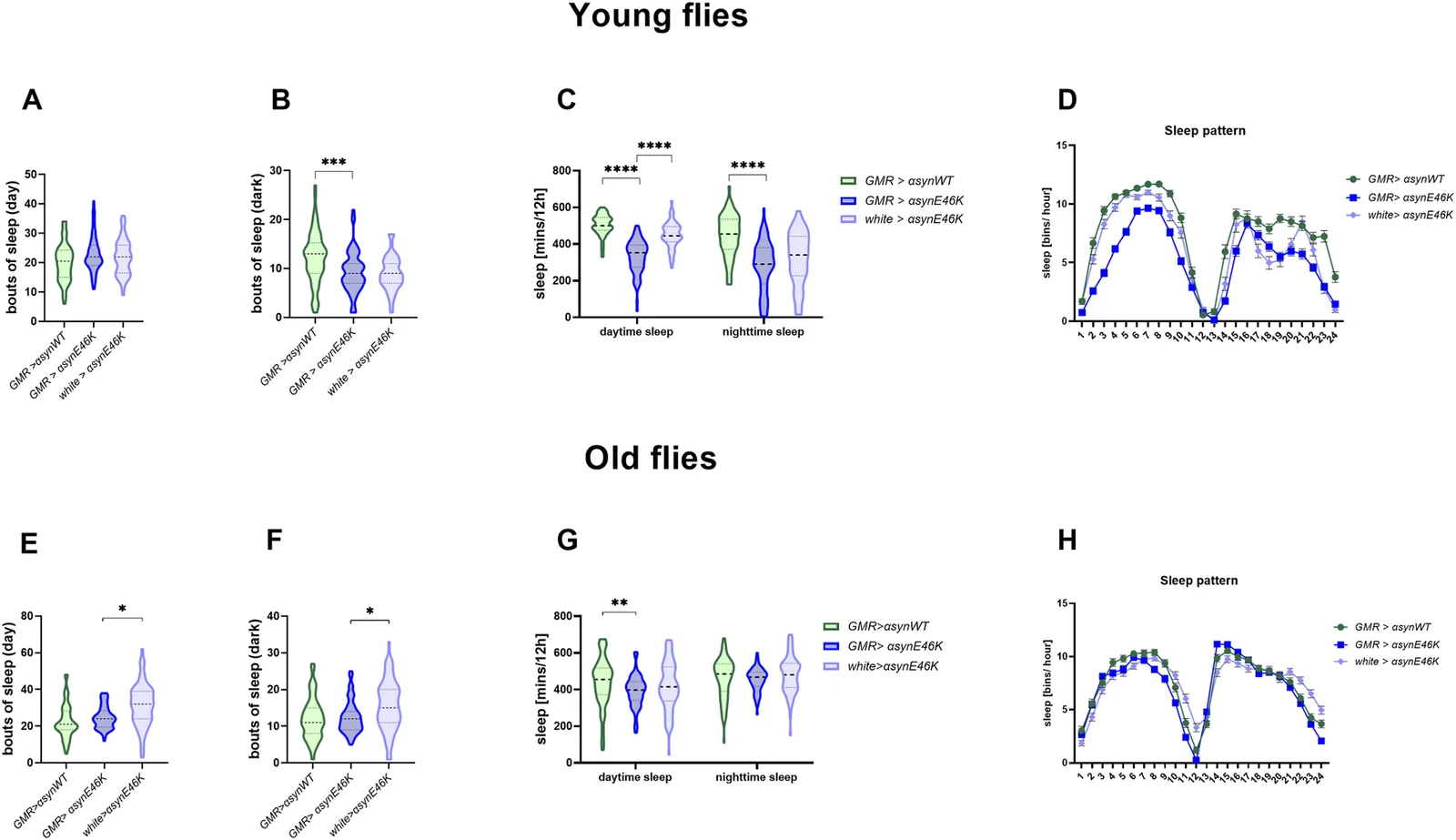
Effect of α-Syn expression in the visual system on sleep homeostasis in young **(A–D)** and old **(E–H)** flies. Sleep fragmentation was observed neither in young **(A,B)** nor in old flies **(E,F)**. Daytime sleep was decreased in young flies **(C)** but not in old individuals **(G)**. Changes in sleep duration were reflected in sleep patterns **(D,H)**. Data represented in median ± IQR. *p < 0.05, **p < 0.01, ****p < 0.0001 (Kruskal–Wallis test with Dunn’s multiple comparison test).

We detected different changes in sleep structure when expressing mutated α-Syn in a specific photoreceptor type in old flies. Analyzing the parameters of sleep episodes, we observed a significant increase in the number of sleep episodes during the day (Figure 9A) but not during the night (Figure 9B) in *Rh1* > *αsynE46K* flies. No changes in the total amount of sleep were detected (Figures 9C,D). Sleep fragmentation was also observed during the day in *Rh3* > *αsynE46K* flies (Figure 9E), but not during the night (Figure 9F). Total nighttime and daytime sleep remained unchanged (Figures 9G,H). For *Rh6* > *αsynE46K* flies there was no effect on sleep episodes (Figures 9I,J), however, they demonstrated a significant increase in the daytime sleep (Figures 9K,L). *Rh1* > *αsynE46K* and *Rh3* > *αsynE46K* exhibited decreased walking activity during the day (Supplementary Figure S11A,B). Moreover *Rh1* > *αsynE46K* showed decreased evening anticipation index, whereas the morning anticipation index of *Rh3* > *αsynE46K* was increased (Supplementary Table S1). Additionally, we also analyzed daily activity profiles (Supplementary Figure S12) and period of locomotor activity (Supplementary Table S1).

**FIGURE 9:**
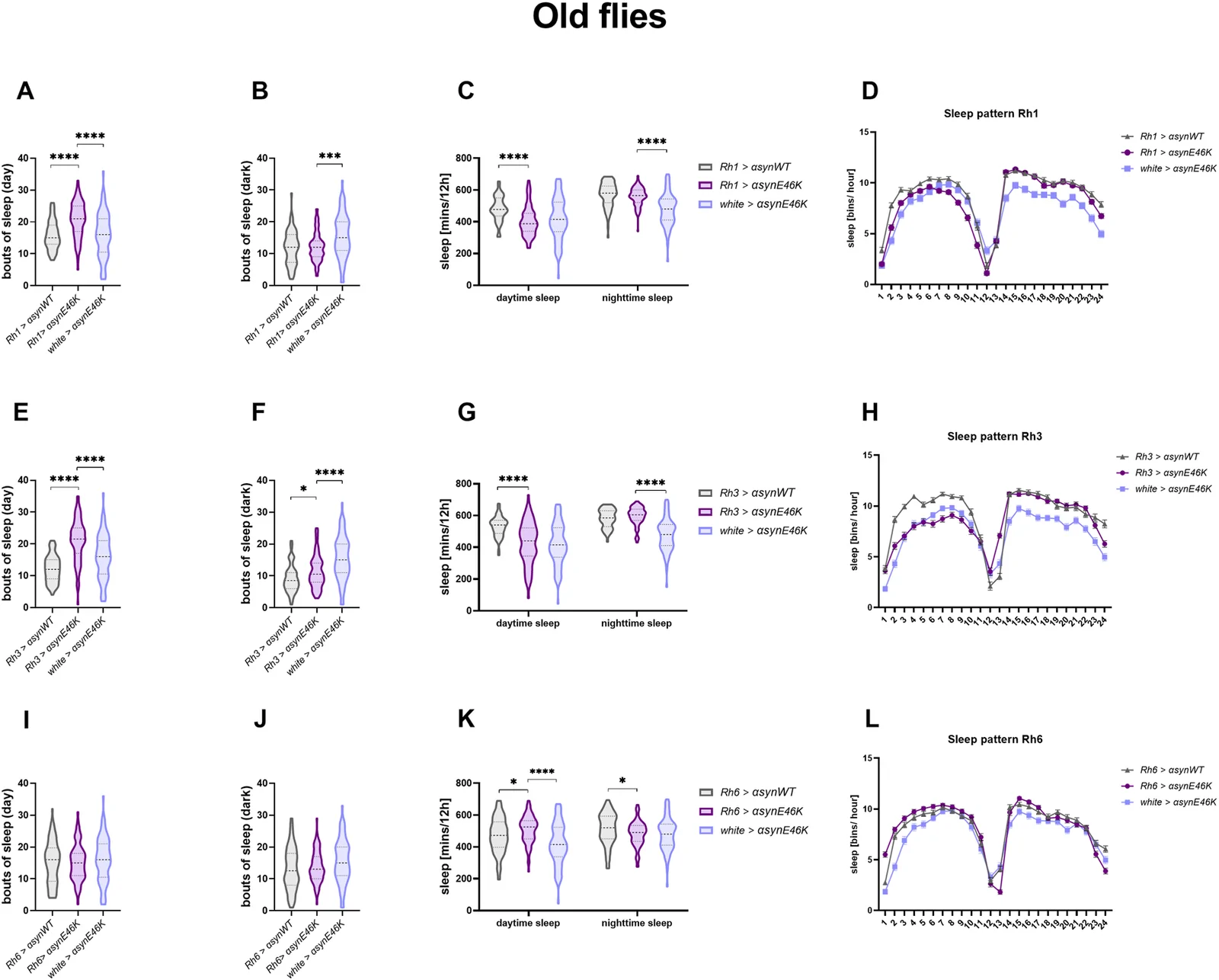
Effect of α-Syn expression in the specific photoreceptor cells on sleep homeostasis in old flies **(A–L)**. Sleep was fragmented during the day in *Rh1* > *αsynE46K* **(A)** and *Rh3* > *αsynE46K* **(E)**, but not in *Rh6* > *αsynE46K* flies **(I)**. Sleep fragmentation was not observed during the night in any of the genotypes **(B,F,J)**. Daytime sleep was increased in *Rh6* > *αsynE46K* **(K)**, but not in *Rh1* > *αsynE46K* **(C)** or *Rh3* > *αsynE46K* **(G)**. Changes in sleep duration were reflected in sleep patterns **(D, H, L)**. Data represented in median ± IQR. *p < 0.05, **p < 0.01, ***p < 0.001, ****p < 0.0001 (Kruskal–Wallis test with Dunn’s multiple comparison test).

#### Effect of α-Syn expression silencing on inflammation, phototransduction and autophagy

The mRNA levels of *Relish* and *Spz* in the retina were elevated in young flies expressing mutated α-Syn (Figure 10A), while in flies expressing wild-type α-Syn we observed a non-significant trend towards increased expression levels. The expression level of *Santa Maria* was decreased in *GMR >* αsynWT (Figure 10B). In the case of the transcriptional alterations in the brain, we observed elevated level of *Relish* in *GMR >* αsynE46K flies (Figure 10D), but no changes in the mRNA levels of *Santa Maria* (Figure 10E) and *Spz* (Figure 10F) were detected. Aged flies expressing wild-type or mutated α-Syn exhibited an increase in mRNA level of *Relish* and *Spz* in the retina, with a significant change observed in wild-type α-Syn expressing flies (Figures 10G,I), but no changes in the expression level of *Santa Maria* were detected (Figure 10H). Moreover, the expression levels of *Relish, Santa Maria* and *Spz* in the brain remained unchanged (Figures 10J–L).

**FIGURE 10:**
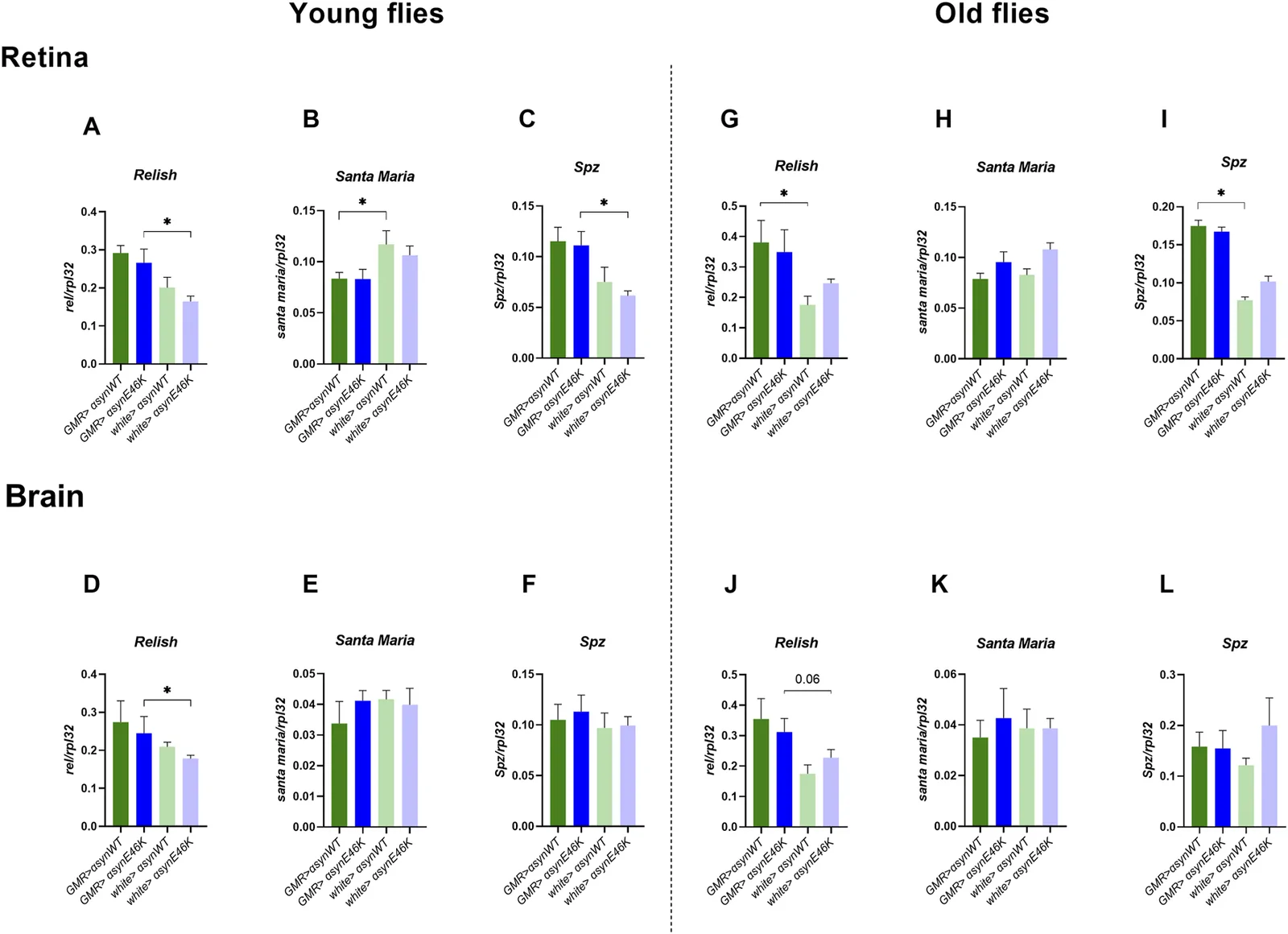
Gene expression analysis in the retina and in the brain of young **(A–F)** and old **(G–L)** flies. Expression levels of *Relish* **(A)** and *Spz* **(C)** were increased in young *GMR >* αsynE46 K flies in the retina. The mRNA level of *Santa maria* was decreased in *GMR >* αsynWT flies **(B)**. Young flies expressing mutated α-Syn exhibited elevated level of *Relish* in the brain **(D)**, with no transcriptional changes in the other genes **(E,F)**. The expression levels of *Relish* **(G)** and *Spätzle* **(I)** in the retina of aged flies were increased in *GMR >* αsynWT, with no change observed in the level of *Santa maria* **(H)**. None of the mRNA levels were altered in the brain **(J–L)**. Results with error bars are means ± SD. *p < 0.05 (Kruskal–Wallis test with Dunn’s multiple comparison test).

We also analyzed the expression levels of *Relish, Spätzle* and *Santia maria* in the retina of flies expressing α-Syn in specific photoreceptor cells (Supplementary Figure S13). Here, the only transcriptional change was observed in *Rh3 > αsynE46K* flies, where the expression of *Relish* was decreased (Supplementary Figure S13B). Additionally, we assessed the mRNA level of *catalase*, both in the retina and brain (Figure 11). No transcriptional alterations were detected in the retina (Figures 11A–C). However, in the brain, expression of *catalase* was decreased in *Rh3* > *αsynE46K* (Figure 11E) and *Rh6* > *αsynE46K* (Figure 11F), but not in *Rh1* > *αsynE46K* (Figure 11D) flies.

**FIGURE 11:**
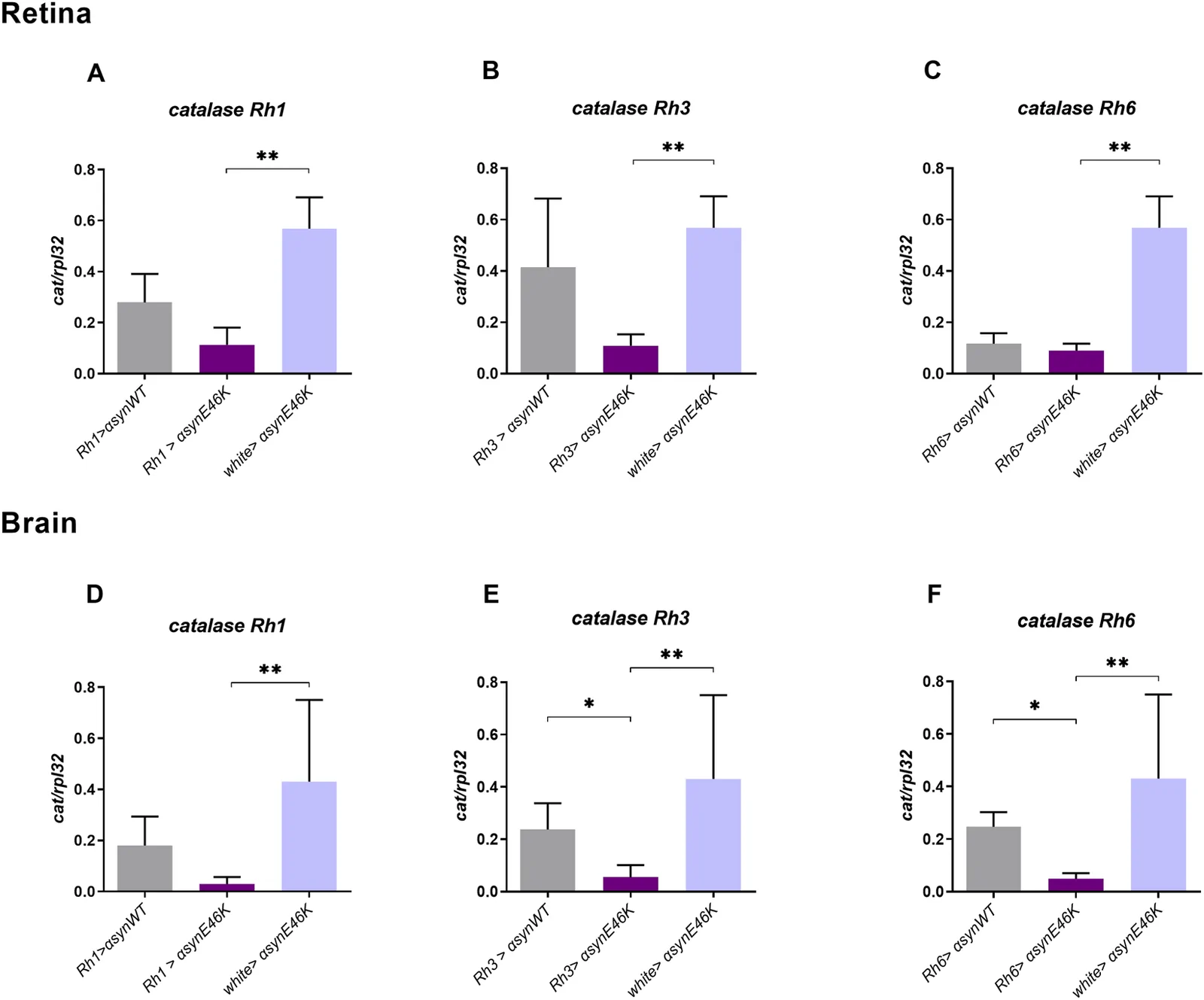
Expression level of *catalase* in the retina **(A–C)** and brain **(D–F)** in aged flies. No significant changes in the expression level of *catalase* were observed in the retina **(A–C)**. The mRNA level of *catalase*, in the brain, was decreased in *Rh3* > *αsynE46K* **(E)** and *Rh6* > *αsynE46K* **(F)**, but not in *Rh1* > *αsynE46K* **(D)** flies. Results with error bars are means ± SD. *p < 0.05, **p < 0.01 (Kruskal–Wallis test with Dunn’s multiple comparison test).

#### Expression of mutated α-Syn reduced the number of interommatidial bristles

We investigated the effect of the expression of mutated α-Syn on the ommatidia structure in young and old flies (Figures 12A–H). We observed the loss of mechanosensory (interommatidial) bristles only in aged *GMR > αsynE46K* flies (Figure 12F), while the ommatidia structure remained unaffected. No changes in the morphology of the *Drosophila* compound eye were detected when expressing α-Syn in a different photoreceptor type (Figures 13A–F).

**FIGURE 12:**
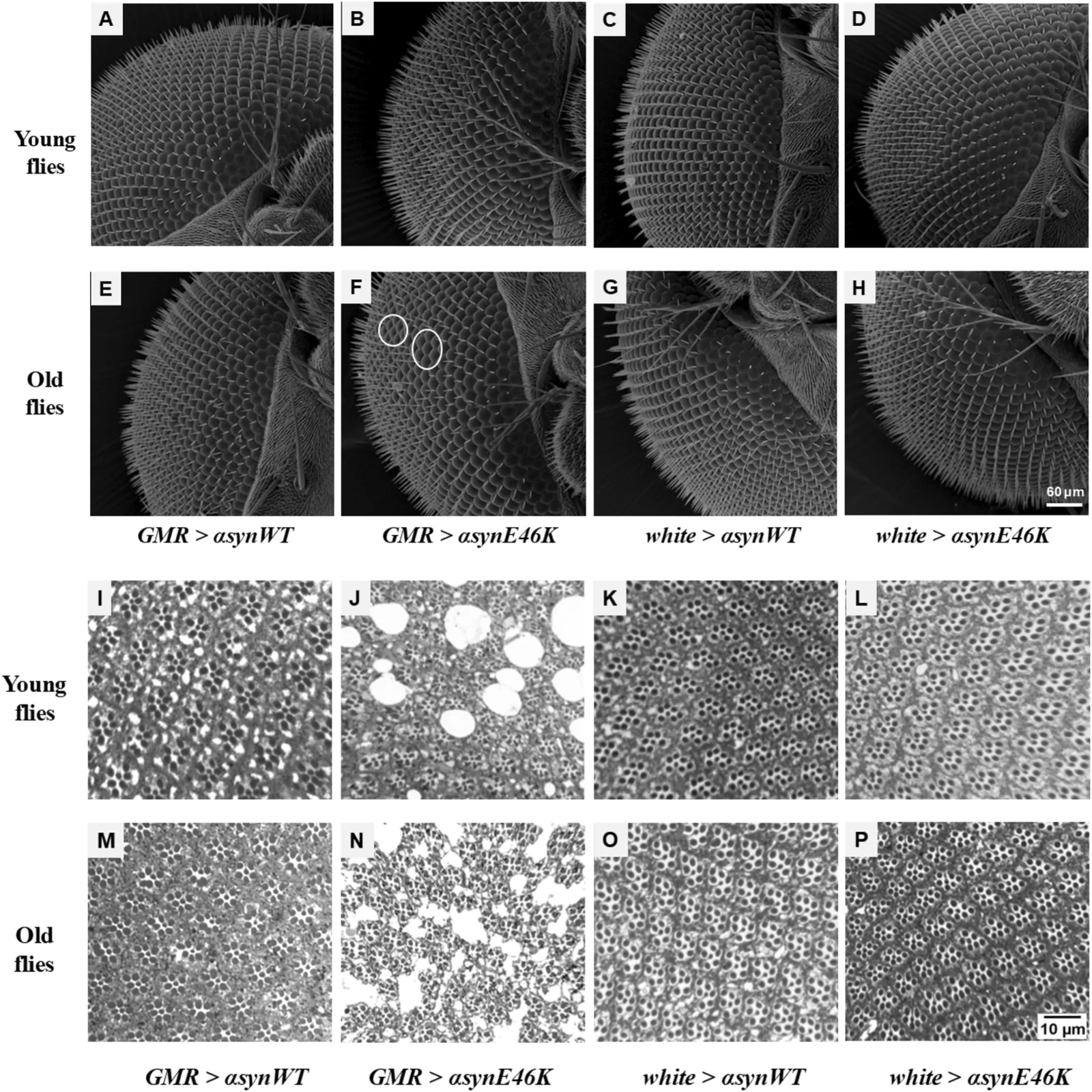
Scanning electron microscopy analysis of the outer eye structures at ×350 magnification **(A–H)**. Expression of mutated α-Syn reduced the number of mechanosensory bristles in the old *GMR > αsynE46K* flies **(F)**. White circles indicate the loss of mechanosensory bristles. Images were obtained from 10 specimens per group. Cross sections of the retina photoreceptors stained with toluidine blue **(I–P)**. Expression of the mutated α-Syn severely distorted the photoreceptors morphology in both young and old flies **(J–N)**. Images were captured from at least three flies per group.

**FIGURE 13:**
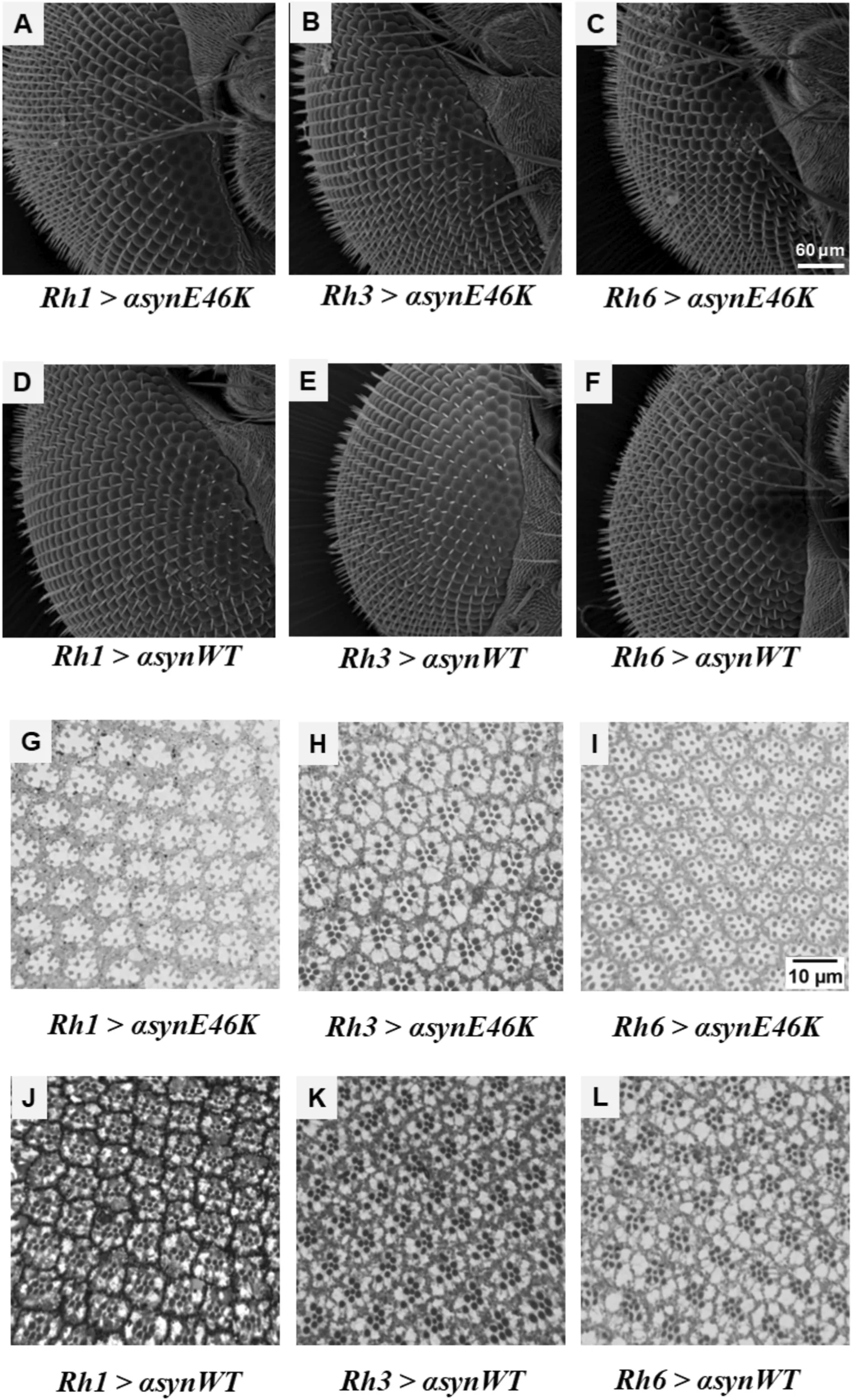
Scanning electron microscopy analysis of the outer eye structures at ×350 magnification **(A–F)**. No changes in the morphology of the *Drosophila* compound eye were observed in any of the genotypes. Cross sections of the retina photoreceptors stained with toluidine blue **(G–L)**. Expression of the mutated α-Syn resulted in smaller rhabdomeres in *Rh1* > *αsynE46K* flies **(G)**. Images were captured from at least three specimens per group.

#### α-Syn disrupts the rhabdomere morphology

To visualize ommatidia organization, we used histological staining with toluidine blue (Figures 12I–P). Cross sections of the retina in *GMR > αsynE46K* flies revealed retinal degeneration in both young (Figure 12J) and old (Figure 12N) individuals, with more severe defects observed in aged flies. Although the photoreceptor rhabdomeres remained visible, the presence of large vacuoles markedly disrupted the ommatidial arrangement. In the case of expressing α-Syn in a specific photoreceptor type (Figures 13G–L), we observed that rhabdomeres in *Rh1* > *αsynE46K* flies (Figure 13G) were prominently smaller than in *Rh3* > *αsynE46K* (Figure 13H) and *Rh6* > *αsynE46K* (Figure 13I), but the trapezoid organization remained unchanged.

#### Expression of α-Syn resulted in degeneration of PAM neurons

α-Syn aggregation mediates dopaminergic neurotoxicity in *Drosophila* models of PD (Feany and Bender, 2000; Periquet et al., 2007). To determine whether α-Syn-induced toxicity in the retina may result in a loss of tyrosine hydroxylase-positive cells in the central brain, we counted neurons in the PAM cluster in both young and old flies (Figure 14). We observed a significant decrease in the number of PAM neurons in aged flies expressing mutated α-Syn (Figure 14B). Interestingly, we also detected PAM cells degeneration upon expressing of aggregated α-Syn in specific photoreceptors (Figure 15). *Rh1* > *αsynE46K* (Figure 15A) and *Rh3* > *αsynE46K* (Figure 15B), but not *Rh6* > *αsynE46K* (Figure 15C) flies exhibited a significant loss of PAM neurons.

**FIGURE 14:**
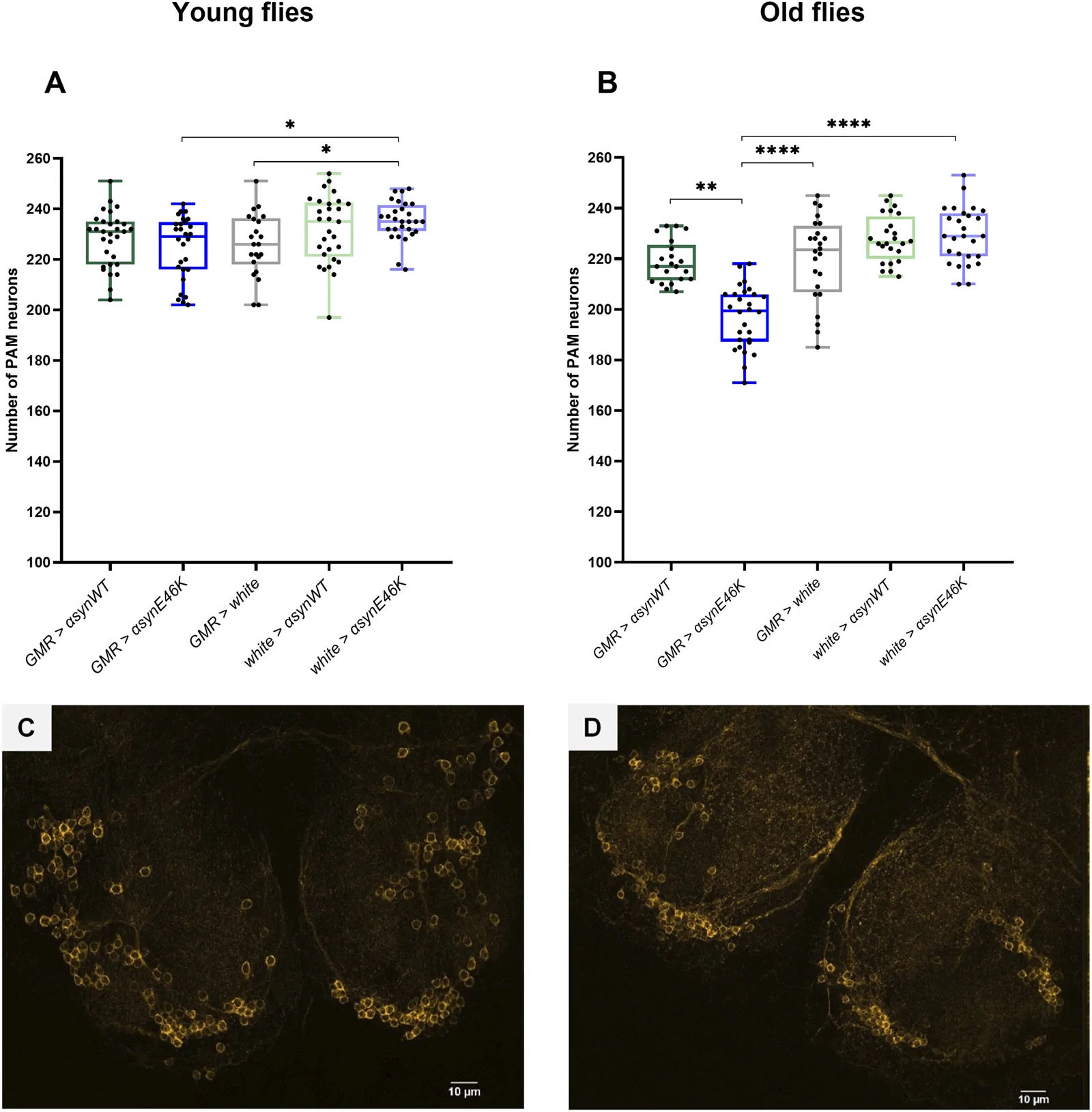
The number of PAM neurons detected by anti-TH immunostaining of young **(A)** and old **(B)** flies. Expression of mutated α-Syn led to PAM cells loss in the old flies compared to the controls **(B)**. Representative maximum intensity projection images of PAM neurons in young **(C)** and old **(D)** *GMR > αsynE46K* flies (242 and 194 cells, respectively). *p < 0.05, **p < 0.01, ****p < 0.0001 (Kruskal–Wallis test with Dunn’s multiple comparison test).

**FIGURE 15:**
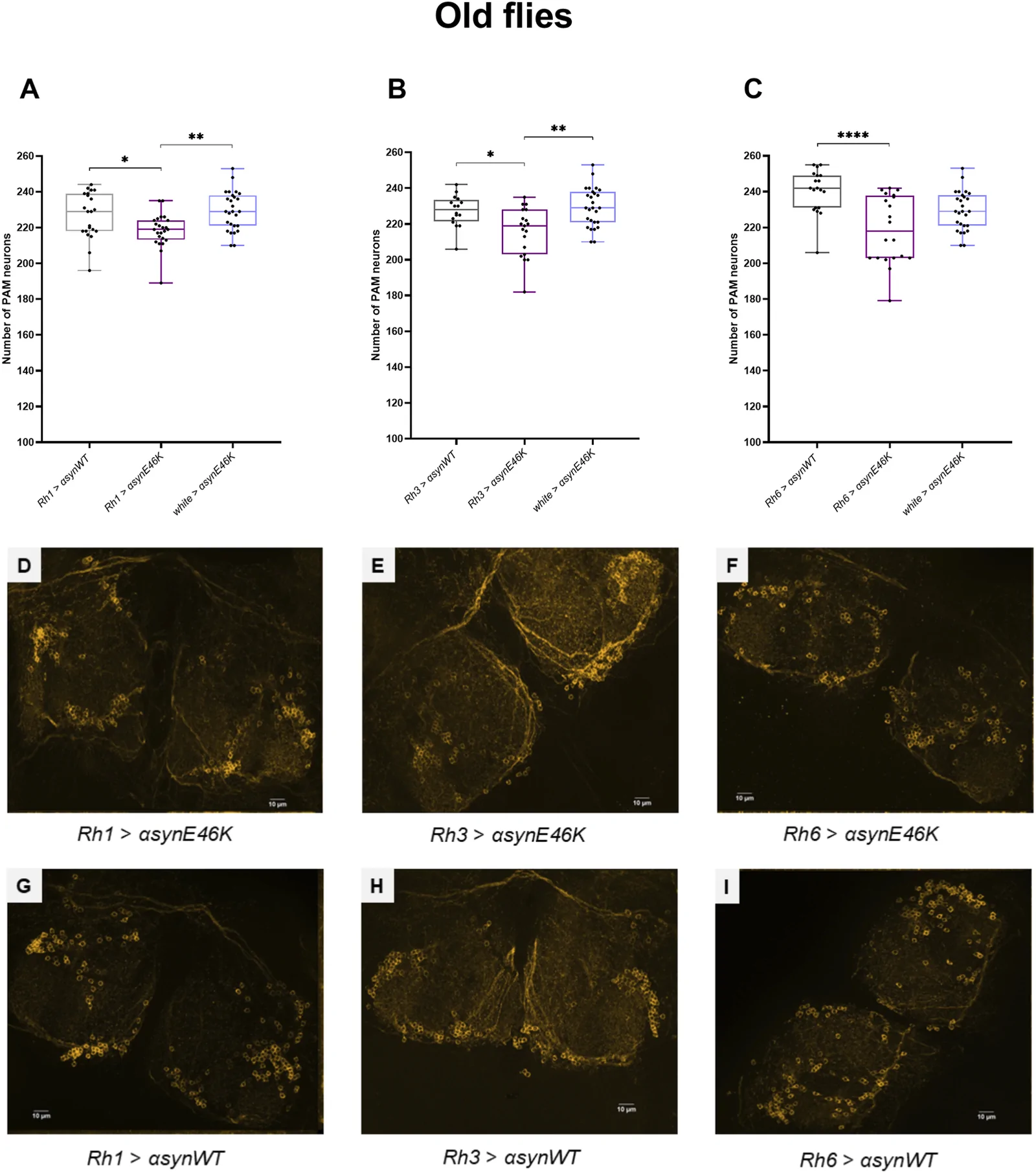
The number of PAM neurons detected by anti-TH immunostaining of old flies **(A–C)**. PAM cells loss was observed in *Rh1* > *αsynE46K* **(A)** and *Rh3* > *αsynE46K* **(B)** but not in *Rh6* > *αsynE46K* flies **(C)**. Representative maximum intensity projection images of PAM neurons **(D–I)**: *Rh1 > αsynE46K* – 214 cells, *Rh3 > αsynE46K* – 217 cells, *Rh6 > αsynE46K* – 238 cells, *Rh1 > αsynWT* – 242 cells, *Rh3 > αsynWT* – 230 cells, *Rh6 > αsynWT* – 250 cells. *p < 0.05, **p < 0.01, ****p < 0.0001 (Kruskal–Wallis test with Dunn’s multiple comparison test).

## Discussion

Parkinson’s disease (PD) is typically recognized as a motor disorder caused by the degeneration of dopaminergic neurons. However, classical motor symptoms, such as tremor and impaired movement, appear relatively late in disease progression. Early manifestations are often non-specific and include olfactory deficits and visual disturbances (Polo et al., 2016; Subramaniam et al., 2023; Orso et al., 2024). Because PD is age-related, declining vision is frequently attributed to normal aging rather than considered an early indicator of neurodegeneration. Importantly, the retina contains dopaminergic neurons, including retinal amacrine cells (Haggendal and Malmfors, 1965), which may degenerate at the early stages of PD (Ortuño-Lizarán et al., 2020). Although the retina is anatomically separated from the brain, being located within the eye, it is an integral part of the nervous system. It maintains functional connections with deeper brain structures. Therefore, cellular degeneration in the retina may influence broader neural circuits, ultimately affecting brain function, behavior, and organismal fitness.

In this study, we used *D. melanogaster* as a model of the genetic form of Parkinson’s disease (PD). Transgenic flies provide a powerful tool to manipulate gene expression in specific cell types. We generated a model in which PD-related alterations, such as disrupted mitophagy or α-Syn aggregation, were restricted to retinal photoreceptors.

The most pronounced effects on brain function were observed when the strong *GMR* driver was used, which enables simultaneous manipulation of all photoreceptor types. However, interpretation of survival and climbing assay results requires caution, as *GMR* exhibits non-specific expression in unidentified neurons in the brain (Ray and Lakhotia, 2015).

In this study, we compared two complementary Parkinson’s disease (PD) models: mitophagy disruption resulting from *parkin* downregulation and synucleinopathy induced by α-Syn expression. By restricting these manipulations to the retina, we established a system to evaluate how degeneration or functional impairment in a defined neuronal population propagates its effects to the central nervous system. This strategy also allowed us to delineate differences in disease progression associated with distinct genetic etiologies of PD.

### Synucleinopathy model

In general, our results showed that flies expressing mutated α-Syn exhibited a reduced lifespan, with increased mortality beginning approximately 22 days after eclosion. Surprisingly, the survival of *GMR>αsyn* flies was only ∼10 days longer than that of *th>α-Syn* flies, expressing α-Syn in dopaminergic cells (Agostini et al., 2023), highlighting how strongly the retina’s health impacts the organism’s condition. Moreover, a link between visual impairment and transneuronal degeneration has been observed previously in humans (Boucard et al., 2009; Garzone et al., 2023). We also detected a significant decrease in the number of PAM neurons in flies with α-Syn expression in the eye. This effect was observed when we used a strong multi-photoreceptor driver, *GMR*, as well as Rhodopsin 1 and Rhodopsin 3 drivers, which allow us to manipulate a specific photoreceptor type; however, we did not see degeneration when using the Rhodopsin 6 driver. In addition, we observed locomotor changes in old flies. Climbing ability was not affected in flies expressing PD-related alterations in R1–R6 photoreceptors, whose axons terminate in the lamina. In contrast, locomotor performance declined in 40-day-old *Rh3>αsynE46K* flies. Importantly, R7/R8-type photoreceptors (including Rh3 and Rh6-expressing subsets) project to deeper neuropils such as the medulla, which are directly involved in higher-order visual processing (Morante and Desplan, 2004). Together, these results indicate that the severity of systemic and behavioral phenotypes correlates with the anatomical depth of photoreceptor projections and their integration into central brain circuits. Disruption of photoreceptors that interface more directly with deeper visual and circadian centers is, therefore, more likely to impair locomotor function and promote neurodegeneration. Consistent with this, we observed pronounced retinal degeneration in flies expressing the E46K mutant form of α-Syn under *GMR*, whereas no severe alterations were detected when expression was restricted using rhodopsin-specific promoters. These findings further support the concept that extensive photoreceptor degeneration can act as a trigger for widespread neuronal dysfunction, potentially via transneuronal mechanisms that propagate pathology to connected brain regions.

To investigate the mechanisms underlying the propagation of degeneration to deeper brain regions, we selected genes associated with autophagy and immune responses and analyzed their expression separately in the retina and brain.

Relish, a homolog of the NF-κB transcription factor, is a key regulator of the Immune deficiency (Imd) pathway, a critical component of the innate immune response. In our experimental flies, we observed elevated *relish* expression in both the retina and brain, suggesting increased production of antimicrobial peptides. Notably, α-Syn induced upregulation of *relish* in these tissues; however, this effect differed between age groups, with mutant α-Syn driving the increase in young flies, whereas wild-type α-Syn did so in old flies. Previous studies have demonstrated that increased Rel expression in glia and chronic activation of immune signaling are linked to neurodegeneration (Petersen and Wassarman, 2012; Cao et al., 2013; Petersen et al., 2013). Furthermore, Rel signaling in photoreceptors regulates non-apoptotic cell death (Chinchore et al., 2012), while in other neuronal populations it can induce toxicity and dendritic abnormalities (Han et al., 2020).

Santa maria, a CD36-like scavenger receptor, is essential for carotenoid transport and subsequent vitamin A production, thereby enabling rhodopsin biosynthesis and maintaining photoreceptor integrity. Disruption of this pathway leads to impaired phototransduction and reduced rhodopsin levels, conditions known to destabilize photoreceptor structure and promote degeneration. In addition, Santa maria contributes to glial phagocytic activity, facilitating the clearance of apoptotic cells and maintaining neural homeostasis; thus, its dysfunction may result in both metabolic stress (due to retinoid deficiency) and impaired debris clearance, creating a permissive environment for neurodegeneration (Wang T. et al., 2007). We observed downregulation of the *santa maria* transcript in young flies expressing wild-type α-Syn, whereas no such changes were detected in flies expressing the mutant form. Interestingly, no alterations were observed in older flies. Given the role of Santa maria in visual function and phagocytic processes, this pattern was unexpected, as impairments in these processes might be anticipated at later stages. However, as *Drosophila* lacks an endogenous α-Syn, ectopic expression of even the wild-type protein may disturb cellular homeostasis, suggesting that the early downregulation reflects an initial transcriptional response rather than sustained, age-dependent dysfunction. Notably, the expression of *santa maria* in the brain remained unchanged.

The third gene analyzed, *spätzle*, plays a dual role in neuronal fitness. Under physiological conditions, Spätzle-mediated activation of the Toll pathway promotes autophagy, a process critical for the clearance of damaged proteins and organelles and for maintaining neuronal survival, particularly in vulnerable populations such as dopaminergic neurons (Zhang et al., 2024). Additionally, Spätzle exhibits neurotrophin-like properties, suggesting that Toll signaling contributes to neuronal plasticity and circuit maintenance. However, chronic or excessive activation of Spätzle signaling can be detrimental. Sustained Toll pathway activation induces innate immune responses, including antimicrobial peptide production and NF-κB-dependent transcription, primarily in glial cells, leading to persistent neuroinflammation (Chowdhury et al., 2019). Such prolonged inflammatory signaling is associated with neuronal dysfunction and degeneration. Consistent with this, we observed increased *spätzle* expression in the retina of young *GMR>αsynE46K* and old *GMR>αsynWT* flies, whereas no changes were detected in the brain, suggesting that inflammatory activation is largely restricted to photoreceptors.

Importantly, our results indicate that both wild-type and mutant α-Syn affect the expression of *relish*, *santa maria*, and *spätzle* similarly, yet lead to distinct phenotypic outcomes. Wild-type α-Syn neither accelerates retinal degeneration nor compromises dopaminergic neuron survival. One possible explanation is that only aggregated forms of α-Syn can propagate pathology or exert toxicity in distal cells. However, a classical seeding mechanism is unlikely in this model, as *Drosophila* lacks endogenous α-Syn expression.

Interestingly, we observed decreased *catalase* mRNA levels in the brain of flies expressing a mutated form of synuclein in the retina, suggesting impairment of systemic antioxidant defenses. This effect is consistent with previous findings demonstrating that α-Syn can directly interfere with antioxidant pathways at the transcriptional level. In particular, experimental studies have shown that α-Syn expression reduces catalase expression and activity in the mouse brain by inhibiting PPARγ-dependent transcription of the catalase gene (Yakunin et al., 2014).

An interesting finding in our data indicates that mutated α-Syn expression in photoreceptors affects sleep architecture. Indeed, recent studies further demonstrate that visual and extraretinal photoreceptors play a direct role in regulating sleep, beyond circadian entrainment. Photoreceptor systems converge onto LNv neurons, which integrate multiple light inputs and mediate light-dependent arousal responses, linking visual processing with sleep–wake regulation. Moreover, genetic perturbations of phototransduction pathways result in altered sleep architecture, including fragmentation of daytime sleep, indicating that proper photoreceptor signaling is required to maintain normal sleep patterns (Au et al., 2023; Hung et al., 2025). In addition, different photoreceptor classes contribute distinctly to sleep regulation: Rh1- and Rh3-expressing cells primarily influence nighttime sleep, whereas Rh5- and Rh6-expressing photoreceptors affect both daytime and nighttime sleep, highlighting the role of deeper-projecting visual circuits in modulating global behavioral states (Damulewicz et al., 2020). These findings are supported by recent findings emphasizing that sleep in *Drosophila* emerges from the interaction between circadian timing, sensory input, including light, and distributed neural circuits (Damulewicz et al., 2020). Notably, Rh6-expressing Hofbauer–Buchner eyelets form synaptic connections with clock neurons (LNv) in the accessory medulla, thereby providing direct photic input to the circadian clock network, which is strongly time-of-day dependent (Veleri et al., 2007; Muraro and Ceriani, 2015; Schlichting et al., 2016; 2019; Damulewicz et al., 2020). Disruption of these pathways may impair light-mediated entrainment of circadian rhythms, leading to dysfunction of the central clock. Given that circadian regulation critically influences locomotor activity and behavioral outputs, such impairments may contribute to the age-dependent changes in sleep architecture observed in experimental flies. Indeed, light signals detected by multiple photoreceptor classes, including retinal and extraretinal structures, play a key role in shaping sleep timing and distribution across the day. Therefore, functional disruption of Rh3- and Rh6-expressing cells may not only disrupt circadian synchronization but also contribute to sleep disturbances, ultimately exacerbating the age-dependent decline in behavioral performance.

These findings are particularly relevant in the context of Parkinson’s disease, in which sleep disturbances are among the earliest and most prevalent non-motor symptoms. In both patients and animal models, circadian disruption and alterations in sleep architecture, including fragmentation, reduced sleep consolidation, and impaired entrainment, are commonly observed before overt motor deficits. Importantly, dopaminergic signaling, which is compromised in PD, is a critical modulator of arousal and sleep–wake transitions (Liu et al., 2012), suggesting a mechanistic link between neurodegeneration and behavioral state regulation.

In this context, degeneration or dysfunction of retinal photoreceptors, especially those projecting to deeper brain regions and directly interfacing with clock neurons, may contribute to PD-related sleep abnormalities by disrupting light-dependent regulation of the circadian system. Loss of proper photic input could impair synchronization of central clock circuits, alter LNv activity, and consequently destabilize sleep–wake rhythms. Such dysfunction is likely to have systemic consequences, as circadian and sleep disturbances can exacerbate neurodegeneration (Dorcikova et al., 2023), impair neuronal homeostasis, and reduce organismal fitness. Indeed, we observed loss of PAM neurons in experimental flies; however, only under some of the drivers.

### Mitophagy disruption

Parkin, encoded by the *PARK2* gene, plays a central role in protecting neurons from degeneration and is therefore a key factor in Parkinson’s disease (PD) development. As an E3 ubiquitin ligase, Parkin is critically involved in mitochondrial quality control, particularly through the PINK1/Parkin-mediated mitophagy pathway, which selectively removes damaged mitochondria (Whitworth and Pallanck, 2017). Loss or downregulation of *parkin* disrupts this process, leading to the accumulation of dysfunctional mitochondria, increased production of reactive oxygen species, and reduced ATP levels, ultimately causing cellular stress and neuronal vulnerability (Doktór et al., 2019). In addition to its role in mitophagy, Parkin contributes to proteostasis by targeting misfolded or toxic proteins for degradation; thus, its deficiency results in proteotoxic stress and exacerbates α-Syn-related pathology (Clausen et al., 2024). These combined defects are particularly detrimental to dopaminergic neurons, which are highly sensitive to metabolic imbalance, leading to the progressive impairment of dopamine release and eventual neuronal loss. Furthermore, Parkin dysfunction has been linked to altered mitochondrial dynamics and enhanced neuroinflammatory signaling, further accelerating neurodegenerative processes (Yan et al., 2023).

Flies with retinal *parkin* silencing exhibited a significantly shortened lifespan; however, the onset of decline was delayed relative to the α-Syn model, beginning at approximately 48 days of age. These animals also displayed reduced climbing ability at 30 days of age, indicating early functional impairment. Notably, no detectable retinal degeneration or loss of dopaminergic neurons was observed, suggesting that *parkin* downregulation in photoreceptors does not directly trigger neurodegeneration in this context. In aged *GMR* > *parkRNAi* flies, sleep was reduced during the daytime and became more fragmented, consistent with circadian disruption. Importantly, the photoreceptor type-specific *parkin* silencing revealed distinct and sometimes opposing effects: in R1–R6 cells, sleep increased and became more consolidated during the night; in Rh3-expressing photoreceptors, daytime sleep was reduced and fragmented, whereas nighttime sleep was elevated; and in Rh6-expressing cells, nighttime sleep was reduced and fragmented. At the molecular level, *santa maria* expression was decreased in the retina, while *relish* and *spätzle* remained unchanged, and no transcriptional alterations were detected in the brain. These findings indicate that *parkin* downregulation primarily affects phototransduction-related processes rather than photoreceptor cell survival. Consequently, the observed behavioral phenotypes, particularly altered sleep architecture, are likely due to destabilized signaling between the visual system and circadian clock circuits. The reduced lifespan in this model may therefore result from chronic sleep disruption rather than from progressive neurodegeneration propagating from the retina to the brain.

Comparison of two PD-related genetic perturbations revealed that they produce distinct phenotypic outcomes. Expression of mutated α-Syn is associated with an earlier onset of mortality and a more rapid decline in survival, indicative of a severe and progressive neurodegenerative process. In contrast, *parkin* silencing leads to a delayed onset of decline, consistent with a gradual accumulation of mitochondrial dysfunction resulting from impaired mitophagy. These differences extend to circadian and sleep regulation. α-Syn aggregation is known to disrupt synaptic transmission and neuronal connectivity, which in the retina is likely to impair signal propagation from photoreceptors to circadian clock circuits. Consequently, this may directly alter LNv neuronal activity, leading to impaired light entrainment, increased circadian desynchronization, and fragmentation of sleep–wake cycles.

In contrast, *parkin*-dependent defects appear to preserve circuit integrity initially but progressively compromise neuronal function through energy imbalance and oxidative stress. In dopaminergic neurons, which are particularly sensitive to mitochondrial dysfunction, this results in a gradual decline in neuronal activity and dopamine availability rather than an acute synaptic failure. When restricted to the retina, mitochondrial impairment in photoreceptors is expected to weaken light-driven input to circadian and dopaminergic circuits over time, producing a delayed but cumulative disruption of circadian regulation and sleep architecture. This model is consistent with the later onset of behavioral decline observed in *parkin* knockdown flies.

Importantly, both mechanisms are likely to converge at the level of dopaminergic modulation of circadian circuits. LNv neurons, which receive input from Rh6-expressing photoreceptors and Hofbauer–Buchner eyelets, are functionally coupled with dopaminergic signaling pathways that regulate arousal and rhythmic behavior. Thus, degeneration or dysfunction of retinal inputs may indirectly alter dopaminergic neuron activity, amplifying systemic dysregulation of sleep–wake cycles.

All together, these findings indicate that α-Syn–induced synaptic dysfunction and *parkin*-dependent mitochondrial impairment differentially affect dopaminergic signaling dynamics, resulting in distinct temporal patterns of circadian and sleep disruption. However, both pathways ultimately converge on impaired dopaminergic regulation of arousal and behavioral rhythms, highlighting the retina as a critical early site linking neurodegeneration with non-motor symptoms of Parkinson’s disease.

## Materials and methods

### *Drosophila* strains

Flies were maintained under LD12:12 conditions (12 h light:12 h dark) at 25 °C on standard cornmeal medium. The following *D. melanogaster* strains were used: *GMR*-Gal4 (Gal4 expressed in all photoreceptor cells), *Rh1*-Gal4 (photoreceptors expressing Rhodopsin 1), *Rh3*-Gal4 (Rhodopsin 3), and *Rh6-*Gal4 (Rhodopsin 6); UAS-*parkRNAi* (dsRNA targeting the *park* gene under UAS control; BDRC 37509); UAS-*αsyn*WT (wild-type human α-Syn under UAS control; BDRC 8146); UAS-*αsynE46K* (human α-Syn with the E46K mutation under UAS control; BDRC 80043); and *w* ^*1118*^ (BDRC 5905). For all experiments, flies were kept under LD12:12 conditions (12 h of light, 12 h of darkness). Unless otherwise indicated, 7-day-old (young) and 40-day-old (old) male flies were used.

### Survival assay

After eclosion, male flies were separated and maintained in groups of 30 individuals, with three replicates per genotype. The number of flies was recorded daily until all individuals in each vial had died. Flies were transferred to fresh food vials every 3 days. Each experiment was repeated three times for each genotype.

### Climbing assay

Males aged 7, 14, 20, and 30 days were transferred into empty vials without the use of CO_2_ to avoid the effects of anesthesia on locomotion. After a short recovery, flies were tapped to the bottom of the vial, and those that climbed vertically beyond the 5 cm marked line within 15 s were counted. The experiment was performed in a dark room illuminated only with red light. Each trial with 30 flies was repeated three times (technical replicates). Each experimental group consisted of 10 biological replicates.

### Sleep analysis and locomotor activity

Locomotor activity was measured using a *Drosophila* Activity Monitoring System (DAMS; Trikinetics). Male flies aged 2 and 30 days were individually placed in glass tubes containing agarose, sucrose, and yeast. The tubes were inserted into monitors equipped with infrared beams that detected activity based on beam interruptions. To assess the circadian period, flies were maintained for 7 days under LD12:12 conditions, followed by 7 days in constant darkness (DD) at 25 °C. Locomotor activity was recorded at 1 min intervals. Sleep was analyzed on the second day of LD12:12 and defined as periods of at least 5 min of inactivity. The morning and evening anticipation indexes were calculated from the activity of flies from 1 day of LD activity. Morning anticipation was calculated as the ratio between the total activity in ZT21-24 and the total activity in ZT 18–24. Evening anticipation was calculated as the ratio between ZT9-12 and ZT6-ZT12.

Data were analyzed in Excel using *Befly!* software (Department of Genetics, University of Leicester). The Lomb–Scargle normalized periodogram was used to identify rhythmic flies; individuals with a periodogram value below 10 (confidence level 0.05) were classified as arrhythmic. To calculate the walking activity, a customized Excel calculator published by another group was used (Chen et al., 2019). Flies that did not survive until the end of each experiment were excluded from the analysis. Each experiment was repeated three times with at least 70 flies per group.

### qPCR

Heads were collected at ZT1 (1 hour after light on), dissected, and both retinal and brain tissues were subjected to total RNA isolation using TRIzol Reagent (Invitrogen) according to the manufacturer’s instructions. RNA concentration and purity were assessed using a NanoDrop spectrophotometer. For reverse transcription, cDNA was synthesized from 500 ng of total RNA using the High Capacity cDNA Reverse Transcription Kit (Thermo Fisher Scientific) with random primers. Quantitative real-time PCR (qRT-PCR) was performed using KAPA SYBR FAST ABI PRISM (Roche) on a StepOnePlus Real-Time PCR System. Primer sequences used for amplification are listed in Table 1. Primer specificity was verified using Primer-BLAST and confirmed by gel electrophoresis. Relative gene expression levels were calculated using the standard curve method, with *RpL32* as the reference gene.

**TABLE 1:** List of primers sequences used in the experiments.

Gene	Sequence
*Rpl32*	F: ATGCTAAGCTGTCGCACAAATG R: GTTCGATCCGTAACCGATGT
*Relish*	F: GGTGATAGTGCCCTGCATGT R: CCATACCCAGCAAAGGTCGT
*santa maria*	F: GGAGGATTTCGGCAACCTTTC R: ATCGGGTCGCTCGATAAAACG
*spätzle*	F: ATCACCACCTGTGACTTCGCC R: GGTCTGCTGTGTGTAGTGCT
*park*	F: TGTGAAAGTCTGGAGGTGGC R: GGAGACATGCTCAGCACACT
*catalase*	F: TTACTGCCACATGAACGGCT R: ATTCTTGATGCCCTGGTCCG

### Immunohistochemistry

Flies were collected at ZT1 and fixed in 4% paraformaldehyde (PFA) for 1 h. Following dissection in PBS, brains were additionally fixed in 4% PFA for 20 min. Next, the brains were washed in PBS for 10 min and three times in 0.2% PBS containing Triton X100 (PBST) for 10 min each. Samples were then incubated in 5% normal goat serum (NGS) in PBS for 30 min at room temperature (RT) for blocking. After blocking, brains were incubated with mouse anti-tyrosine hydroxylase primary antibody (1:1,000, ImmunoStar) for 2 days at 4 °C. Subsequently, the samples were washed three times in 0.2% PBST for 10 min each and incubated for 2 h at RT with goat anti-mouse Cy3-conjugated secondary antibody (1:250, Abcam). Finally, brains were washed three times in 0.2% PBST and once in PBS for 10 min each, and whole brains were mounted in Vectashield medium (Vector).

### Fluorescence microscopy and image analysis

Fly brains were imaged using a Zeiss Axio Imager two microscope. To quantify PAM dopaminergic neurons, Z-stacks were acquired and cell segmentation was performed using a pretrained model from the Cellpose library (v4.1.1 (Stringer et al., 2021)). Segmentation masks were inspected in Fiji/ImageJ by overlaying regions of interest (ROIs) and using the Cell Counter plugin for visualization and manual curation. Cellpose was run on individual optical slices (2D) and the resulting masks were subsequently stitched into 3D in Fiji by merging ROIs corresponding to the same cell across adjacent slices based on centroid overlap (i.e., 2D masks were linked across z-planes using spatial overlap criteria). All samples were reviewed by the investigator, and segmentation masks were corrected as needed by adding or removing ROIs. Supplementary Figure S14 shows an example of Cellpose segmentation for a representative optical slice.

### Scanning electron microscopy

To examine changes in the external morphology of the *Drosophila* eye, scanning electron microscopy (SEM) was performed. Fly heads were fixed overnight in 2.5% glutaraldehyde in cacodylate buffer. The following day, samples were washed four times for 15 min each in cacodylate buffer, dehydrated through a graded ethanol series, and subsequently transferred to pure acetone. Specimens were dried using CO_2_ critical point drying and coated with gold. Imaging was carried out using a JEOL JSM-5410 scanning electron microscope.

### Histological toluidine blue staining

To investigate photoreceptor degeneration in the retina, histological staining was performed. Fly heads, with the proboscis removed, were fixed for 2 h in a solution containing 2% glutaraldehyde and 2.5% paraformaldehyde in 0.3 M cacodylate buffer supplemented with CaCl_2_. Samples were then washed in 0.1 M cacodylate buffer for 15 min and post-fixed for 1 h in 1% OsO_4_ in veronal acetate buffer containing CaCl_2_ and sucrose. Subsequently, tissues were dehydrated through an ethanol series followed by propylene oxide and embedded in Poly/Bed 812 resin (Polysciences). Semi-thin retinal sections (∼500 nm) were cut using a Leica EM UC7 ultramicrotome and stained with toluidine blue. Images were acquired using a Zeiss Axio Imager two microscope.

### Statistical analysis

GraphPad Prism (version 8.0.1) was used for statistical analysis. Normal distribution of residuals was assessed using the Shapiro-Wilk test. To evaluate the variance homogeneity the Barlett’s or Brown-Forsythe test was used. Outliers were identified using the ROUT test. For data meeting the assumptions of normality and homogeneity of variance, one-way analysis of variance (ANOVA) with *post hoc* Tukey’s multiple comparison test was used. To compare non-normally distributed data, the Kruskal–Wallis one-way ANOVA with Dunn’s multiple comparison test was used. The statistical significance for all experiments was set at *p* < 0.05. Violin plots show the distribution of the data, with the median indicated by a dashed line and the interquartile range (IQR; 25th to 75th percentiles) indicated by dotted lines.

## Data Availability

The datasets presented in this study can be found in online repositories. The names of the repository/repositories and accession number(s) can be found below: https://doi.org/10.57903/UJ/F93RDW.
