# Supplementary material for: From retina to brain: retinal dysfunction and dopaminergic neurodegeneration in Drosophila models of Parkinson’s disease
Source: Front Cell Dev Biol. 2026 Sep 23;14:1929128. doi: 10.3389/fcell.2026.1929128 (PMC13643024; doi:10.3389/fcell.2026.1929128)
Supplement: Supplementary file 1 [file Table1.docx]

Table S1.

Locomotor activity, and morning and evening anticipation index of flies.

| **Age** | **Genotype** | **Period of the rhythm [h]** | **Morning anticipation index** | **Evening anticipation index** | **Number of flies** |
| --- | --- | --- | --- | --- | --- |
| **Mitophagy disruption model** | | | | | |
| Young flies | *GMR > parkRNAi* | 23.66 ± 0.46 | 0,59 ± 0.12 | 1.00 ± 0.00 | 66 |
|  | *GMR > white* | 23.59 ± 0.67 | 0,65 ± 0.14 | 1.00 ± 0.00 | 84 |
|  | *white > parkRNAi* | 23.18 ± 0.49 | 0,63 ± 0.18 | 1.00 ± 0.00 | 89 |
| Old flies | *GMR > parkRNAi* | 24.33 ± 0.44 | 0.71 ± 0.14 | 0.91 ± 0.092 | 75 |
|  | *GMR > white* | 24.25 ± 0.41 | 0.68 ± 0.18 | 0.92 ± 0.11 | 100 |
|  | *white > parkRNAi* | 23.79 ± 0.64 | 0.55 ± 0.17 | 0.85 ± 0.16 | 96 |
| Old flies | *Rh1 > parkRNAi* | 24.27 ± 0.37 | 0,62 ± 0.28 | 0.86 ± 0.15 | 90 |
|  | *Rh3 >  parkRNAi* | 24.29 ± 0.64 | 0,67 ± 0.22 | 0.77 ± 0.15 | 93 |
|  | *Rh6 >  parkRNAi* | 23.75 ± 0.46 | 0,58 ± 0.17 | 0.73 ± 0.15 | 84 |
|  | *Rh1 > white* | 24.02 ± 0.29 | 0,66 ± 0.17 | 0.85 ± 0.11 | 96 |
|  | *Rh3 > white* | 23.84 ± 0.39 | 0,62 ± 0.17 | 0.91 ± 0.08 | 108 |
|  | *Rh6 > white* | 23.98 ± 0.36 | 0,65 ± 0.13 | 0.79 ± 0.11 | 89 |
| **synucleinopathy model** | | | | | |
| Young flies | *GMR > αsynWT* | 23.55 ± 0.31 | 0.65 ± 0.20 | 1.00 ± 0.00053 | 83 |
|  | *GMR > αsynE46K* | 23.72 ± 0.43 | 0.62 ± 0.12 | 0.80 ± 0.13 | 104 |
|  | *white > αsynWT* | 23.13 ± 0.55 | 0.70 ± 0.16 | 0.97 ± 0.046 | 107 |
|  | *white > αsynE46K* | 22.82 ± 0.46 | 0.68 ± 0.12 | 0.86 ± 0.14 | 82 |
| Old flies | *GMR > αsynWT* | 24.36 ± 0.60 | 0.69 ± 0.18 | 0.92 ± 0.093 | 82 |
|  | *GMR > αsynE46K* | 24.13 ± 0.34 | 0.69 ± 0.12 | 0.83 ± 0.11 | 76 |
|  | *white > αsynWT* | 24.01 ± 0.48 | 0.64 ± 0.21 | 0.91 ± 0.10 | 80 |
|  | *white > αsynE46K* | 24.22 ± 0.51 | 0.64 ± 0.19 | 0.81 ± 0.16 | 85 |
| Old flies | *Rh1> αsynE46K* | 23.66 ± 0.32 | 0.69 ± 0.17 | 0.75 ± 0.15 | 92 |
|  | *Rh3 > αsynE46K* | 23.96 ± 0.37 | 0.76 ± 0.19 | 0.69 ± 0.20 | 82 |
|  | *Rh6> αsynE46K* | 23.74 ± 0.39 | 0.69 ± 0.16 | 0.82 ± 0.15 | 91 |
|  | *Rh1 > αsynWT* | 23.79 ± 0.35 | 0.70 ± 0.22 | 0.83 ± 0.15 | 80 |
|  | *Rh3 > αsynWT* | 24.03 ± 0.56 | 0.59 ± 0.28 | 0.92 ± 0.09 | 62 |
|  | *Rh6 > αsynWT* | 24.00 ± 0.41 | 0.68 ± 0.20 | 0.80 ± 0.17 | 104 |
|  |  |  |  |  |  |
